# Partially non-homogeneous dynamic Bayesian networks based on Bayesian regression models with partitioned design matrices

**DOI:** 10.1093/bioinformatics/bty917

**Published:** 2018-11-05

**Authors:** Mahdi Shafiee Kamalabad, Alexander Martin Heberle, Kathrin Thedieck, Marco Grzegorczyk

**Affiliations:** 1 Department of Mathematics, Bernoulli Institute, Faculty of Science and Engineering, University of Groningen, AG Groningen, The Netherlands; 2 Laboratory of Pediatrics, Section Systems Medicine of Metabolism and Signaling, University of Groningen, University Medical Center Groningen, AV Groningen, The Netherlands; 3 Department for Neuroscience, School of Medicine and Health Sciences, Carl von Ossietzky University Oldenburg, Oldenburg, Germany

## Abstract

**Motivation:**

Non-homogeneous dynamic Bayesian networks (NH-DBNs) are a popular modelling tool for learning cellular networks from time series data. In systems biology, time series are often measured under different experimental conditions, and not rarely only some network interaction parameters depend on the condition while the other parameters stay constant across conditions. For this situation, we propose a new partially NH-DBN, based on Bayesian hierarchical regression models with partitioned design matrices. With regard to our main application to semi-quantitative (immunoblot) timecourse data from mammalian target of rapamycin complex 1 (mTORC1) signalling, we also propose a Gaussian process-based method to solve the problem of non-equidistant time series measurements.

**Results:**

On synthetic network data and on yeast gene expression data the new model leads to improved network reconstruction accuracies. We then use the new model to reconstruct the topologies of the circadian clock network in *Arabidopsis thaliana* and the mTORC1 signalling pathway. The inferred network topologies show features that are consistent with the biological literature.

**Availability and implementation:**

All datasets have been made available with earlier publications. Our Matlab code is available upon request.

**Supplementary information:**

Supplementary data are available at *Bioinformatics* online.

## 1 Introduction

Dynamic Bayesian networks (DBNs) have become a popular tool for learning the topologies of cellular regulatory networks from time series data. The classical (homogeneous) DBN models assume that the network parameters stay constant in time, so that the network structure is inferred along with one single set of network parameters (Friedman *et al.*, 2000). Many regulatory processes are non-stationary so that this homogeneity assumption is too restrictive. To allow for time-dependent parameters, many authors have proposed to combine DBNs with multiple changepoint processes (Ahmed and Xing, 2009; Dondelinger *et al.*, 2013; Grzegorczyk and Husmeier, 2013; Husmeier *et al.*, 2010; Lèbre *et al.*, 2010; Robinson and Hartemink, 2010) or with hidden Markov models (Grzegorczyk, 2016). In those models a multiple changepoint process (or a hidden Markov model) divides the temporal data into disjoint components with component-specific network parameters. The network structure, the data segmentation and the component-specific network parameters are inferred from the data. Those models are often referred to as non-homogeneous DBNs (NH-DBNs).

In many real-world applications, in particular in systems biology, data are often collected under different experimental conditions. That is, instead of one single (long) time series that has to be segmented, there are *K* (short) time series. The data are then intrinsically divided into *K* unordered components, and there is no need for inferring the segmentation. In this situation, it is normally not clear a priori whether the network parameters stay constant across components (conditions) or whether they vary from component to component (with the conditions). Three biological systems that we will consider in this article are: Section 4.4: The parameters of a metabolism-related regulatory network in *Saccharomyces cerevisiae* can depend on the medium, in which yeast is cultured (glucose versus galactose). Section 4.5: The parameters of the circadian clock network in *Arabidopsis thaliana* can depend on the dark: light cycles, to which the plants were earlier exposed. Section 4.6: The parameters of the mammalian target of rapamycin complex 1 (mTORC1) protein signalling network can change in the presence of insulin. For more examples and a thorough discussion on the integration of single-cell data from multiple experimental conditions we refer to Geissen *et al.* (2016).

If the parameters stay constant, all data can be merged and analysed with one single homogeneous DBN. If the parameters are component-specific, then the data should be analysed by a NH-DBN. The disadvantage of both approaches is that all parameters are assumed to be either constant (DBN) or component-specific (NH-DBN). In real-world applications there can be both types of parameters, so that both models are inappropriate. For example, if a variable *Y* is regulated by two other variables, $X_{1}\to Y\leftarrow X_{2}$, then the interaction $X_{1}\to Y$ can stay constant, while $X_{2}\to Y$ might be component-specific, e.g. for *K* = 2 and in terms of a regression model:

(1) $$E[Y|X_{1}=x_{1},X_{2}=x_{2}]=\{\begin{array}{cc} \alpha x_{1}+\beta x_{2} & \;\text{if}\;k=1\\ \alpha x_{1}+\gamma x_{2} & \;\text{if}\;k=2 \end{array}$$

A DBN ignores that β and γ are different. A NH-DBN has to infer the same parameter α two times separately. Both model misspecifications can increase the inference uncertainty, and are thus critical for sparse data.

No tailor-made model for the situation in (1) has been proposed yet. To fill this gap, we propose a partially NH-DBN model, which infers the best trade-off between a DBN and a NH-DBN. Unlike all earlier proposed NH-DBNs, the new partially NH-DBN model operates on the individual interactions (network edges). For each interaction there is a parameter, and the model infers from the data whether the parameter is constant or component-specific. We implement the new model in a hierarchical Bayesian regression framework, since this model class reached the highest network reconstruction accuracy in the cross-method comparison by Aderhold *et al.* (2014). But we note that the underlying idea is generic and could also be implemented in other frameworks, e.g. via L1-regularized regression models (‘LASSO’). 

In Section 2.5 we propose a Gaussian process (GP)-based method to deal with the problem of non-equidistant measurements. The standard assumption for all NH-DBNs is that data are measured at equidistant time points. For applications where this assumption is not fulfilled, we propose to use a GP to predict the values at equidistant data points and to replace the non-equidistant values by predicted equidistant values. We will make use of the GP method when analysing the mTORC1 data in Section 4.6.

## 2 Materials and methods

DBNs and NH-DBNs are used to infer networks showing the regulatory interactions among variables $Z_{1},\ldots ,Z_{N}$. The interactions are subject to a time lag, so that there is no need for an acyclic network structure. Hence, dynamic network inference can be thought of as inferring the covariate sets for *N* independent regression models. In the *i*th model, *Z_i_* is the response and the remaining $N_{*}:=N-1$ variables $Z_{1},\ldots ,Z_{i-1},Z_{i+1},\ldots ,Z_{N}$ at time point *t* − 1 are used as potential covariates for *Z_i_* at time point t. The goal is to infer a covariate set for each *Z_i_*, and the system of covariate sets describes a network; see Section 2.6 for details. As the same regression model is applied to each *Z_i_* separately, we describe it using a general notation, where *Y* is the response and $X_{1},\ldots ,X_{n}$ are the covariates.

### 2.1 Bayesian regression with partitioned design matrix

We consider a regression model with response *Y* and covariates $X_{1},\ldots ,X_{n}$. We assume that data were measured under *K* experimental conditions, which we refer to as *K* components. We further assume that the data for each component k∈{1,…,K} were measured at equidistant time points $t=1,\ldots ,T_{k}$. Let $y_{k,t}$ and $x_{i,k,t}$ denote the values of *Y* and *X_i_* at the *t*th time point of component *k*. In dynamic networks, the interactions are subject to a time lag O, which is usually set to one time point. That is, the values $x_{1,k,t},\ldots ,x_{n,k,t}$ correspond to the response value $y_{k,t+1}$. For each component *k* we build a component-specific response vector $y_{k}$ and the corresponding design matrix $X_{k}$, where $X_{k}$ includes a first column of 1’s for the intercept:

$$y_{k}={\left(y_{k,2},\ldots ,y_{k,T_{k}}\right)}^{T},\;\;\;\;X_{k}=\left(\begin{array}{cccc} 1 & x_{1,k} & \ldots & x_{n,k} \end{array}\right)$$

where $x_{i,k}={\left(x_{i,k,1},\ldots ,x_{i,k,T_{k}-1}\right)}^{T}$

For each *k* we could assume a separate Gaussian likelihood:

(2) $$y_{k}∼N_{T_{k}-1}(X_{k}\beta_{k},\sigma_{k}^{2}I)\;\;\;\;\;\;(k=1,\ldots ,K)$$

where I is the identity matrix, $\beta_{k}={\left(\beta_{k,0},\beta_{k,1},\ldots ,\beta_{k,n}\right)}^{T}$ is the component-specific vector of regression coefficients, and $\sigma_{k}^{2}$ is the component-specific noise variance. Imposing independent priors on each pair $\left\{\beta_{k},\sigma_{k}^{2}\right\}$, leads to *K* independent models. Alternatively, we could merge the data $y:={\left(y_{1}^{T},\ldots y_{K}^{T}\right)}^{T}$ and $X:={\left(X_{1}^{T},\ldots ,X_{K}^{T}\right)}^{T}$ and employ one model for the merged data:

(3) $$y∼N_{T}(X\beta ,\sigma^{2}I)\;\;\;\;\text{where}\;\;T:=\sum_{k=1}^{K}\left(T_{k}-1\right)$$

so that $\beta ={\left(\beta_{0},\beta_{1},\ldots ,\beta_{n}\right)}^{T}$ would apply to all components.

When some covariates have a component-specific and other covariates have a constant regression coefficient, both likelihoods (2) and (3) are suboptimal. For this situation, we propose a new partially non-homogeneous regression model that infers the best trade-off from the data. The key idea is to use a likelihood with a partitioned design matrix.

For now, we assume that we know for each coefficient whether it is component-specific or constant. Let the intercept and the first $n_{1}<n$ coefficients stay constant while the remaining $n_{2}=n-n_{1}$ coefficients are component-specific. We then have the regression equation:

$$y_{k,t+1}=\beta_{0}+\sum_{\text{i}=1}^{n_{1}}\beta_{i}\cdot x_{i,k,t}+\sum_{i=n_{1}+1}^{n}\beta_{k,i}\cdot x_{i,k,t}+\epsilon_{k,t+1}$$

where $\epsilon_{k,t+1}\;∼N(0,\sigma^{2})$, and the likelihood takes the form:

(4) $$y∼N_{T}(X_{B}\beta_{B},\sigma^{2}I)$$

where $\beta_{B}$ is a vector of $\left(1+n_{1}+K\cdot n_{2}\right)$ regression coefficients, and $X_{B}$ is a partitioned matrix with $T=\sum \left(T_{k}-1\right)$ rows and $\left(1+n_{1})+(K\cdot n_{2}\right)$ columns. For example, for *K* = 2 the matrix $X_{B}$ has the structure:

$$\left(\begin{array}{cccccccccc} 1 & x_{1,1} & \ldots & x_{n_{1},1} & x_{n_{1}+1,1} & \ldots & x_{n,1} & 0 & \ldots & 0\\ 1 & x_{1,2} & \ldots & x_{n_{1},2} & 0 & \ldots & 0 & x_{n_{1}+1,2} & \ldots & x_{n,2} \end{array}\right),$$

where $x_{i,k}={\left(x_{i,k,2},\ldots ,x_{i,k,T_{k}-1}\right)}^{T}$, and $\beta_{B}$ is of the form:

$${\left((\beta_{0},\beta_{1},\ldots ,\beta_{n_{1}}),(\beta_{n_{1}+1,1},\ldots ,\beta_{n,1}),(\beta_{n_{1}+1,2},\ldots ,\beta_{n,2})\right)}^{T}$$

The first subvector of $\beta_{B}$ is the vector $\beta_{*}:={\left(\beta_{0},\beta_{1},\ldots ,\beta_{n_{1}}\right)}^{T}$ of the regression coefficients that stay constant, and then there is a subvector $\beta_{k}:={\left(\beta_{n_{1}+1,k},\ldots ,\beta_{n,k}\right)}^{T}$ for each component *k* with the component-specific regression coefficients. For the noise variance parameter $\sigma^{2}$ we use an inverse Gamma prior, $\sigma^{-2}∼\text{Gam}(a,b)$, and on $\beta_{*}$ we impose a Gaussian prior with zero mean vector:

(5) $$\beta_{*}∼N_{n_{1}+1}(0,\sigma^{2}\lambda_{*}^{2}I)$$

For the component-specific vectors $\beta_{1},\ldots ,\beta_{K}$ we adapt the idea from Grzegorczyk and Husmeier (2013), and impose a hyperprior:

(6) $$\beta_{k}∼N_{n_{2}}(\mu ,\sigma^{2}\lambda_{⋄}^{2}I)\;\;\;(k=1,\ldots ,K)\;\;\;\;\text{and}\;\;\;\;\mu ∼N_{n_{2}}(\mu_{0},\Sigma_{0})$$

The hyperprior couples the vectors $\beta_{1},\ldots ,\beta_{K}$ hierarchically and encourages them to stay similar across components. Re-using the variance parameter $\sigma^{2}$ in (5 and 6) allows the regression coefficient vectors and the noise variance to be integrated out in the likelihood, i.e. the marginal likelihood $p(y|\lambda_{*}^{2},\lambda_{⋄}^{2},\mu )$ to be computed analytically (see below). For $\lambda_{*}^{2}$ and $\lambda_{⋄}^{2}$ we also use inverse Gamma priors:

$$\lambda_{*}^{-2}∼\text{Gam}(\alpha_{*},\beta_{*})\;\text{and}\;\lambda_{⋄}^{-2}∼\text{Gam}(\alpha_{⋄},\beta_{⋄})$$

The prior of $\beta_{B}={\left(\beta_{*}^{T},\beta_{1}^{T},\ldots ,\beta_{K}^{T}\right)}^{T}$ is a product of Gaussians:

$$p(\beta_{B}|\sigma^{2},\lambda_{⋄}^{2},\lambda_{*}^{2},\mu )=p(\beta_{*}|\sigma^{2},\lambda_{*}^{2})\cdot \prod_{k=1}^{K}p(\beta_{k}|\sigma^{2},\lambda_{⋄}^{2},\mu )$$

Given $\sigma^{2},\;\lambda_{⋄}^{2},\;\lambda_{*}^{2}$, and μ, the Gaussians are independent, so that:

$$\beta_{B}|(\sigma^{2},\lambda_{⋄}^{2},\lambda_{*}^{2},\mu )∼N_{1+n_{1}+K\cdot n_{2}}(\tilde{\mu },\sigma^{2}\tilde{\Sigma })$$

$$\text{with}:\;\;\tilde{\mu }={\left(0^{T},\mu^{T},\ldots ,\mu^{T}\right)}^{T}\;\;\;\text{and}\;\;\;\tilde{\Sigma }=\left(\begin{array}{cc} \lambda_{*}^{2}I_{*} & 0\\ 0 & \lambda_{⋄}^{2}I_{⋄} \end{array}\right)$$

where $I_{*}$ is the $\left(n_{1}+1\right)$-dimensional and $I_{⋄}$ the $\left(K\cdot n_{2}\right)$-dimensional identity matrix. We have for the posterior distribution:

(7) $$p(\beta_{B},\sigma^{2},\lambda_{*}^{2},\lambda_{⋄}^{2},\mu |y)\propto p(y|\sigma^{2},\beta_{B})\cdot p(\beta_{B}|\sigma^{2},\lambda_{⋄}^{2},\lambda_{*}^{2},\mu )\ldots \ldots \cdot p(\mu )\cdot p(\sigma^{-2})\cdot p(\lambda_{*}^{-2})\cdot p(\lambda_{⋄}^{-2})$$

**Fig. 1.**
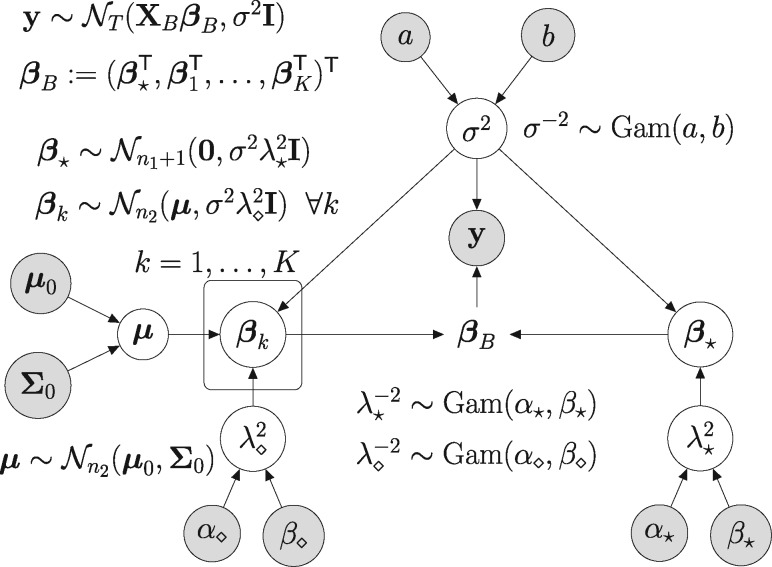
Graphical model representation of the regression model with partitioned design matrix. Variables that have to be inferred are in white circles. The data and the fixed hyperparameters are in grey circles. The vector $\beta_{B}$ deterministically depends on $\beta_{*}$ and $\beta_{1},\ldots ,\beta_{K}$. The vector $\beta_{k}$ in the plate is condition-specific

A graphical model representation of the new regression model is provided in Figure 1. The full conditional distributions of $\beta_{B},\;\sigma^{2},\;\lambda_{*}^{2},\;\lambda_{⋄}^{2}$ and μ can be computed analytically, so that Gibbs-sampling can be applied to generate a posterior sample. As the derivations are mathematically involved, we relegate them to Part A of the Supplementary Material.

The marginalization rule from Section 2.3.7 of Bishop (2006) yields:

(8) $$p(y|\lambda_{⋄}^{2},\lambda_{*}^{2},\mu )=\frac{\Gamma \left(\frac{T}{2}+a\right)}{\Gamma (a)}\cdot \frac{\pi^{-\frac{T}{2}}{\left(2b\right)}^{a}}{\det{\left(C\right)}^{1/2}}\cdot \ldots \ldots \cdot {\left(2b+{\left(y-X_{B}\tilde{\mu }\right)}^{T}C^{-1}(y-X_{B}\tilde{\mu })\right)}^{-\left(\frac{T}{2}+a\right)}\text{where}\;T:=\sum_{k=1}^{K}\left(T_{k}-1\right),\;\;\text{and}\;C:=I+X_{B}\tilde{\Sigma }X_{B}^{T}.$$

### 2.2 Inferring the relevant covariates and their types

In typical applications, there is a set of $N_{*}$ variables, and the subset of the relevant covariates has to be inferred from the data. Each covariate can be either constant (δ = 1) or component-specific (δ = 0). Let $\Pi =\{X_{1},\ldots ,X_{n}\}$ be a subset of the $N_{*}$ variables, and let $\delta ={\left(\delta_{0},\delta_{1},\ldots ,\delta_{n}\right)}^{T}$ be a vector of binary variables, where δ_i_ indicates whether *X_i_* has a constant ($\delta_{i}=1$) or component-specific ($\delta_{i}=0$) regression coefficient. The first element, δ_0_, refers to the intercept.

The goal is then to infer the covariate set Π and the corresponding indicator vector δ from the data. For any combination of Π and δ, the partitioned design matrix $X_{B}=X_{B,\Pi ,\delta }$ can be built, and the marginal likelihood $p(y|\lambda_{⋄}^{2},\lambda_{*}^{2},\mu ,\Pi ,\delta )$ can be computed with (8). We get for the posterior:

$$\begin{array}{c} p(\Pi ,\delta ,\lambda_{*}^{2},\lambda_{⋄}^{2},\mu |y)\propto p(y|\lambda_{⋄}^{2},\lambda_{*}^{2},\mu ,\Pi ,\delta )\cdot p(\Pi )\cdot p(\delta |\Pi )\cdot \ldots \ldots \cdot p(\mu |\Pi ,\delta )\cdot p(\lambda_{*}^{2})\cdot p(\lambda_{⋄}^{2}) \end{array}$$

where p(μ|Π,δ) is a Gaussian, whose dimension is the number of component-specific coefficients. For the covariate sets, Π, we follow Grzegorczyk and Husmeier (2013) and assume a uniform distribution, truncated to |Π|≤3. The prior p(δ|Π) will be specified in Section 2.5. To generate samples from the posterior, we use a Markov Chain Monte Carlo (MCMC) algorithm, which combines the Gibbs-sampling steps for $\beta_{B},\;\sigma^{2},\;\lambda_{*}^{2},\;\lambda_{⋄}^{2}$ and μ with two blocked Metropolis Hastings (MHs) moves. In the first MH move the vector δ is sampled jointly with μ, and in the second MH move Π is sampled jointly with δ and μ. As the implementation of the MCMC algorithm is involved, we relegate the mathematical details to Parts B and C of the Supplementary Material.

### 2.3 Competing models

A homogeneous model merges all data, while a non-homogeneous model assumes each component *k* to have specific parameters; see (2). The new partially non-homogeneous model infers the best trade-off: Each regression coefficient can be either constant or component-specific.

For a fair comparison, we also allow the non-homogeneous model to switch between a homogeneous and a non-homogeneous state. However, like all models that have been proposed so far, it operates on the covariate sets. All covariates have either component-specific (*S* = 0) or constant (*S* = 1) regression coefficients. In our method comparison, we include:

- DBN: A homogeneous model that merges all data, see (3).
- NH-DBN: The NH-DBN model switches between two states. We have a DBN for *S* = 1, and the likelihood takes the form of (2) for *S* = 0.
- coupled NH-DBN: This model from Grzegorczyk and Husmeier (2013) is an NH-DBN that globally couples the regression coefficients.

### 2.4 Specifying the covariate type prior

The NH-DBNs can switch between: ‘all covariates are constant’ (*S* = 1) versus ‘all covariates are component-specific’ (S = 0). Those states refer to δ=1 and δ=0 of the partially NH-DBN. To match the priors, we set:

(9) $$\frac{p(S=1)}{p(S=0)}=\frac{p(\delta =1|\Pi )}{p(\delta =0|\Pi )}$$

For $\Pi =\{X_{1},\ldots ,X_{n}\},\;\delta$ contains *n* + 1 binary elements, which we assume to be independently Bernoulli distributed. To fulfil (9) the Bernoulli parameter must depend on n=|Π|. We get: $p(\delta =1|\Pi )=\theta_{n}^{n+1}$ and $p(\delta =0|\Pi )={\left(1-\theta_{n}\right)}^{n+1}$. From (9) we obtain:

$$\begin{array}{c} r:=\frac{p(S=1)}{p(S=0)}=\frac{\theta_{n}^{n+1}}{{\left(1-\theta_{n}\right)}^{n+1}}\Leftrightarrow \theta_{n}={\left(\frac{r}{1+r}\right)}^{1/(n+1)}\text{and}\;\;p(\delta |\Pi )=\theta_{n}^{\sum_{i=0}^{n}\delta_{i}}\cdot {\left(1-\theta_{n}\right)}^{\sum_{1=0}^{n}\left(1-\delta_{i}\right)} \end{array}$$

For mixture models it is often assumed that the number of components $\tilde{K}$ has a Poisson distribution (Green, 1995). We truncate it to $\tilde{K}\in \{1,K\}$:

$$p(S=0)=\frac{q(K)}{q(1)+q(K)}\text{ and }p(S=1)=\frac{q(1)}{q(1)+q(K)}$$

where q(.) is the density of the Poisson distribution with parameter θ = 1.

### 2.5 GP smoothing for non-equidistant data

The regression models assume that the time lag O between the response value $y_{k,t+1}$ and the covariate values $x_{1,k,t},\ldots ,x_{n,k,t}$ is the same for all *t*. If the data within a component *k* were measured at time points $t_{1},\ldots ,t_{T_{k}}$, with varying distances $O_{i}:=t_{i}-t_{i-1}$, the models lead to biased results. For this scenario, we propose to replace the observed non-equidistant response values by predicted equidistant response values. We propose the following GP-based method:

- Determine the lowest time lag $O^{*}=\min\{O_{2},\ldots ,O_{T_{k}}\}$, where $O_{i}:=t_{i}-t_{i-1}$.
- Given the observed data points $\left\{(t,y_{k,t}):t=t_{1},\ldots ,t_{T_{k}}\right\}$, use a GP to predict the whole curve ${\left\{(t,y_{k,t})\right\}}_{t\geq 0}$.
- Extract the response values at the time points: $t_{1}+O^{*},\ldots ,t_{T_{k}}+O^{*}$.
- Build the response vector and design matrix such that the values $x_{1,k,t_{i}},\ldots ,x_{n,k,t_{i}}$ are used to explain the predicted response value $y_{k,t_{i}+O^{*}}$ ($i=1,\ldots ,T_{k}$). The new lag is then constant; $O_{t}=O^{*}$.

A GP is a stochastic process ${\left\{Y_{k,t}\right\}}_{t\geq 0}$, here indexed by time, such that every finite subset of the random variables has a Gaussian distribution. A GP can be used to estimate a non-linear curve ${\left(t,y_{k,t}\right)}_{t\geq 0}$ from noisy observations. We here assume the relationship: $y_{k,t}=f(t)+\epsilon_{t}$ where $\epsilon_{t}∼N(0,\sigma^{2})$ is observational noise, and the non-linear function f(.) is unknown. We estimate f(.) by fitting a GP to the observed data. The GP defines a distribution over the functions f(.), which transforms the input ($t_{1},\ldots ,t_{T_{k}})$ into output ($y_{k,t_{1}},\ldots ,y_{k,t_{T_{K}}})$, such that

(10) $${\left(Y_{k,t_{1}},\ldots ,Y_{k,t_{T_{K}}}\right)}^{T}∼N_{T_{k}}(0,K+\sigma^{2}I)$$

where I is the identity matrix, and the elements of the *T_k_*-by-*T_k_* covariance matrix, K, are defined through a kernel function: $K_{i,j}=\xi^{2}\cdot k(t_{i},t_{j})$ with signal variance parameter $\xi^{2}$. The kernel function k(.,.) is typically chosen such that similar inputs *t_i_* and *t_j_* yield correlated variables $Y_{t_{i}}$ and $Y_{t_{j}}$. A popular and widely used kernel is the squared exponential kernel with: $k(t_{i},t_{j})=\text{exp}\;\left(-\frac{1}{2}\cdot \frac{{\left(t_{i}-t_{j}\right)}^{2}}{l^{2}}\right)$ where *l* is the length scale. For the unobserved vector $y_{k,*}:={\left(y_{k,t_{1}+O^{*}},\ldots ,y_{k,t_{T_{k}}+O^{*}}\right)}^{T}$ we then have the predictive distribution:

(11) $$y_{k,*}∼N({\hat{y}}_{k,*},{\hat{\Sigma }}_{k,*})$$

with

(12) $$\begin{array}{l} {\hat{y}}_{k,*}:=(K^{*}+\sigma^{2}I)\cdot {\left(K+\sigma^{2}I\right)}^{-1}\cdot y\\ {\hat{\Sigma }}_{k,*}:=(K^{**}+\sigma^{2}I)-(K^{*}+\sigma^{2}I){\left(K+\sigma^{2}I\right)}^{-1}{\left(K^{*}+\sigma^{2}I\right)}^{T} \end{array}$$

where $y:={\left(y_{k,t_{1}},\ldots ,y_{k,t_{T_{K}}}\right)}^{T}$ is the observed response vector, and $K^{*}$ and $K^{**}$ are *T_k_*-by-*T_k_* matrices, whose elements are given by: ${\left(K^{*}\right)}_{i,j}:=\xi^{2}\cdot k(t_{i}+O^{*},t_{j})$ and ${\left(K^{**}\right)}_{i,j}:=\xi^{2}\cdot k(t_{i}+O^{*},t_{j}+O^{*})$. Before inferring the GP, we standardize y to mean 0, and we impose log-uniform priors on the GP parameters ($\sigma^{2},\;\xi^{2}$ and *l*). For predicting the unobserved response vector, we have to make two decisions:

1. The GP parameters can either be sampled via MCMC simulations or their maximum a posteriori (MAP) estimates can be computed.
2. Given GP parameters, the vector $y_{k,*}$ can be sampled from (11) or it can be set equal to its predictive expectation, ${\hat{y}}_{k,*}$, defined in (12).

We have implemented and cross-compared all four combinations. For lack of space, we here report the results obtained for predictive expectations based on MAP estimates. A comparison of the four approaches can be found in Part D of the Supplementary Material.

### 2.6 Learning topologies of regulatory networks

Assume that the variables $Z_{1},\ldots ,Z_{N}$ interact with each other in form of a network and that data were collected under *K* conditions and that the conditions influence some of the interactions. Let $D_{k}$ denote the *N*-by-*T_k_* data matrix which was measured under condition *k*. The rows of $D_{k}$ correspond to the variables and the columns of $D_{k}$ correspond to *T_k_* time points. $D_{k,i,t}$ denotes the value of *Z_i_* at time point *t* under condition *k*.

The goal is to infer the network structure. Interactions for temporal data are usually modelled with a time lag, e.g. of order O=1. An edge, $Z_{j}\to Z_{i}$, indicates that *Z_j_* has an effect on *Z_i_* in the following sense: For all *k* the value $D_{i,k,t+1}$ (*Z_i_* at *t* + 1) depends on $D_{j,k,t}$ (*Z_j_* at *t*).

There is no acyclicity constraint, and DBN inference can be thought of as inferring *N* separate regression models and combining the results. In the *i*th model $Y:=Z_{i}$ is the response. The remaining $N_{*}:=N-1$ variables $Z_{1},\ldots ,Z_{i-1},Z_{i+1},\ldots ,Z_{N}$ are the potential covariates. For each $Y:=Z_{i}$ we infer a covariate set $\Pi_{i}$, and the covariate sets $\Pi_{1},\ldots ,\Pi_{N}$ describe a network N. There is the edge $Z_{j}\to Z_{i}$ in the network N if and only if $Z_{j}\in \Pi_{i}$.

We can thus apply the partially non-homogeneous model to each *Y* = *Z_i_* separately, to generate posterior samples. We extract the covariate sets, $\Pi_{i}^{\left(1\right)},\ldots ,\Pi_{i}^{\left(R\right)}$ (i=1,…,N), and we merge them to a network sample $N^{\left(1\right)},\ldots ,N^{\left(R\right)}$. The *r*th network $N^{\left(r\right)}$ possesses the edge $Z_{j}\to Z_{i}$ if and only if $Z_{j}\in \Pi_{i}^{\left(r\right)}$. For each edge $Z_{j}\to Z_{i}$ we can then estimate its marginal posterior probability (‘score’):

$${\hat{s}}_{j,i}=\frac{1}{R}\sum_{r=1}^{R}I_{j\to i}(N^{\left(r\right)})\;\;\;\text{where}\;\;\;I_{j\to i}(N^{\left(r\right)})=\{\begin{array}{cc} 1 & \;\text{if}\;Z_{j}\in \Pi_{i}^{\left(r\right)}\\ 0 & \;\text{if}\;Z_{j}\notin \Pi_{i}^{\left(r\right)} \end{array}$$

When the true network is known, we can evaluate the network reconstruction accuracy with precision-recall curves. For each ψ∈[0,1] we extract the n(ψ) edges whose scores ${\hat{s}}_{j,i}$ exceed ψ, and we count the number of true positives T(ψ) among them. Plotting the precisions P(ψ):=T(ψ)/n(ψ) against the recalls R(ψ):=T(ψ)/M, where *M* is the number of edges in the true network, gives the precision–recall curve (Davis and Goadrich, 2006). We refer to the area under the curve as AUC value. The higher the AUC, the higher the reconstruction accuracy.

## 3 Implementation

For the inverse Gamma distributed parameters ($\sigma^{2},\;\lambda_{*}^{2},\;\lambda_{⋄}^{2}$) we use shape and rate parameters from earlier works, e.g. in Grzegorczyk and Husmeier (2013) and Lèbre *et al.* (2010): $\sigma^{-2}∼\text{Gam}(0.005,0.005)$ and $\lambda_{*}^{-2},\lambda_{⋄}^{-2}∼\text{Gam}(2,0.2)$ and for the hyperprior on μ we use $\mu_{0}=0$ and $\Sigma_{0}=I$. Other settings led to comparable results what indicates robustness w.r.t. those hyperparameters. To ensure a fair comparison we use the same hyperparameters for the competing models; cf. Section 2.3.

For generating posterior samples, we run the MCMC algorithm from Section 2.2 for 100 000 (100k) iterations. We set the burn-in phase to 50k and we sample every 100th graph during the sampling phase. This yields *R* = 500 posterior samples for each response *Y* = *Z_i_*. We merge the individual covariate sets $\Pi_{i}^{\left(r\right)}$ (i=1,…,N; r=1,…,R) to a network sample $N^{\left(1\right)},\ldots ,N^{\left(R\right)}$, as explained in Section 2.6. For each edge $Z_{j}\to Z_{i}$ we then compute its edge score ${\hat{s}}_{j,i}$.

We used scatter plots of edge scores from independent simulations to monitor convergence. In Section 4.3 we study convergence, scalability and the computational costs for model inference.

We implement the GP method with the squared exponential kernel and used the Matlab package ‘GPstuff’ (Vanhatalo *et al.*, 2013) to numerically determine the MAP estimates of the parameters via scaled conjugate gradient optimization. We also tested other kernels, such as the Matern 3/2 and 5/2 kernel, and for them we obtained very similar results.

## 4 Empirical results

### 4.1 Pre-study 1: GP smoothing

Our first objective is to provide empirical evidence that the proposed GP method from Section 2.5 can yield substantial improvements. To this end, we generate values for 10 autoregressive (AR) variables:

(13) $$X_{i,t}=\sqrt{1-\eta }\cdot X_{i,t-1}+\sqrt{\eta }\cdot \epsilon_{i,t}\;\;\;(t=0,\ldots ,120;i=1,\ldots ,10)$$

where all $\epsilon_{i,t}$’s are independently *N*(0, 1) distributed, $X_{i,1}∼N(0,1)$ for all *i*, and η∈(0,1). This yields: $X_{i,t}∼N(0,1)$ for all *t* and all *i*. We further assume that *X*_1_ and *X*_2_ are covariates for:

(14) $$Y_{t+1}=\beta_{0}+\beta_{1}X_{1,t}+\beta_{2}X_{2,t}+\epsilon_{y,t+1}$$

where $\epsilon_{y,t+1}∼N(0,0.01^{2})$.

In a second scenario we replace (13) by moving averages (MA):

(15) $$X_{i,t}=\sum_{j=t-q}^{t}\epsilon_{i,j}\;\;\;(t=0,1,\ldots ,120;i=1,\ldots ,10)$$

where all $\epsilon_{i,t}$’s are independently $N(0,{\left(q+1\right)}^{-1})$ distributed, so that again $X_{i,t}∼N(0,1)$ for all *i* and *t*.

**Fig. 2.**
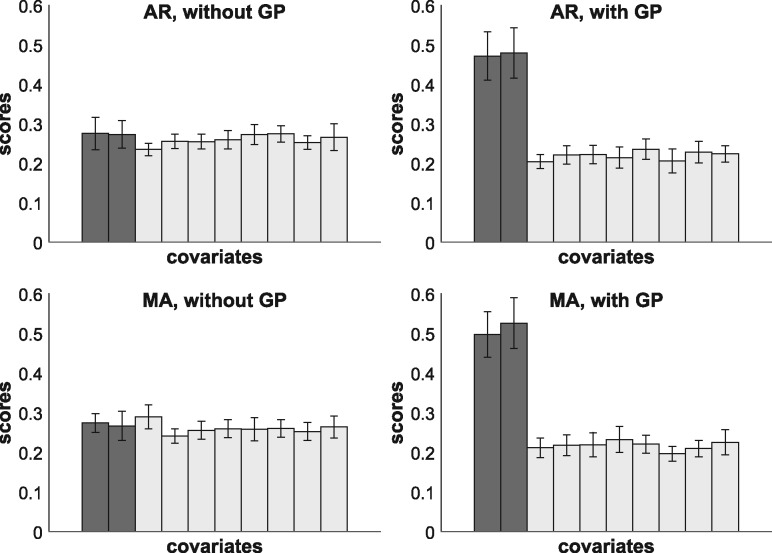
Average scores (posterior probabilities). In each histogram, the dark grey bars refer to the scores of the true covariates, and the light grey bars refer to the irrelevant variables. Covariate values were generated via AR (top) and MA processes (bottom). The left histograms show the scores of a standard regression (without GP processing). The right histograms show the scores when the proposed GP method is used. Error bars indicate SDs

We generate data for both scenarios (AR and MA) with different parameter settings $\left(\beta_{0},\beta_{1},\beta_{2}\right)$ in (14) and η in (13), respective *q* in (15). We thin the data out and keep only the observations at the time points t∈{0,1,3,5,10,15,30,45,60,120}, as the same time points were measured for the mTORC1 data; see Section 4.6. The standard regression approach uses the covariate values at *t_i_* for explaining *Y* at $t_{i+1}$, although the time lag steadily increases. The GP method from Section 2.5 predicts the response values at $t_{i}+O^{*}$, and replaces $y_{t_{i+1}}$ (observed *Y* at $t_{i+1}$) by ${\hat{y}}_{t_{i}+O^{*}}$ (predicted *Y* at $t_{i}+O^{*}$), where $O^{*}=1$.With both approaches we run MCMC simulations on each dataset, and from the MCMC samples we compute for each covariate *X_i_* the score that *X_i_* is a covariate for *Y*. Our results show that the proposed GP method finds the true covariates *X*_1_ and *X*_2_, while the standard approach cannot clearly distinguish them from the irrelevant variables $X_{3},\ldots ,X_{8}$. Figure 2 shows histograms of the average covariate scores for AR data with $\beta_{i}=1$ and η=0.2, and for MA data with $\beta_{i}=1$ and *q* = 10.

### 4.2 Pre-study 2: Synthetic RAF-pathway data

The RAF pathway, as reported in Sachs *et al.* (2005), consists of *N* = 11 nodes and 20 directed edges. The topology of the RAF pathway is shown in Part E of the Supplementary Material. We generate data with *K* = 2 components and *T_k_* = 10 data points each. The parent nodes of each node *Z_i_* build its covariate set $\Pi_{i}$. We assume a linear model with component-specific regression coefficients:

$$z_{i,k,t+1}=\beta_{k,0}^{i}+\sum_{j:Z_{j}\in \Pi_{i}}\beta_{k,j}^{i}\cdot z_{j,k,t}+e_{k,t}^{i}\;\;\;(k=1,2)$$

where $z_{i,k,t}$ denotes the value of node *Z_i_* at time point *t* in component *k*, and $\beta_{k,j}^{i}$ is the regression coefficient for $Z_{j}\to Z_{i}$ in component *k*. The noise values $e_{k,t}^{i}$ and the initial values $z_{i,k,1}$ are sampled from independent $N(0,0.05^{2})$ distributions. For *Z_i_* there are $2(|\Pi_{i}|+1)$ component-specific regression coefficients. For each Z_i_ we collect them in two vectors $\beta_{k}^{i}$ (*k* = 1, 2), and we sample the elements of $\beta_{k}^{i}$ from *N*(0, 1) Gaussian distributions. We then re-normalize the vectors to Euclidean norm one: $\beta_{k}^{i}\leftarrow \beta_{k}^{i}/|\beta_{k}^{i}|$ (*k* = 1, 2). We distinguish six scenarios:

- (S1) Identical: We withdraw $\beta_{2}^{i}$ and assume that the same regression coefficients apply to both components. We set: $\beta_{2}^{i}=\beta_{1}^{i}$ for all *i*.
- (S2) Identical signs (correlated): We enforce the coefficients to have the same signs, i.e. we replace $\beta_{2,j}^{i}$ by: $\beta_{2,j}^{i}:=\text{sign}(\beta_{1,j}^{i})\cdot |\beta_{2,j}^{i}|$ for all *i* and *j*.
- (S3) Uncorrelated: We use the vectors $\beta_{k}^{i}$ for component *k* (*k* = 1, 2). The component-specific coefficients $\beta_{1,j}^{i}$ and $\beta_{2,j}^{i}$ are then uncorrelated for all *i* and all *j*.
- (S4) Opposite signs (negatively correlated): We withdraw the vector $\beta_{2}^{i}$ and we set: $\beta_{2,j}^{i}=(-1)\cdot \beta_{1,j}^{i}$. The coefficients $\beta_{1,j}^{i}$ and $\beta_{2,j}^{i}$ are then negatively correlated.
- Mixture of (S1) and (S3): We assume that 50% of the coefficients are identical for both *k*, while the other 50% are uncorrelated. We randomly select 50% of the coefficients and set: $\beta_{2,j}^{i}=\beta_{1,j}^{i}$. The other 50% of the coefficients stay unchanged (uncorrelated).
- Mixture of (S1) and (S4): We withdraw $\beta_{2}^{i}$ and we assume that 50% of the coefficients are identical for both *k*, while the other 50% have an opposite sign. We randomly select 50% of the coefficients and set: $\beta_{2,j}^{i}=\beta_{1,j}^{i}$. For the other coefficients we set $\beta_{2,j}^{i}=(-1)\cdot \beta_{1,j}^{i}$.

**Fig. 3.**
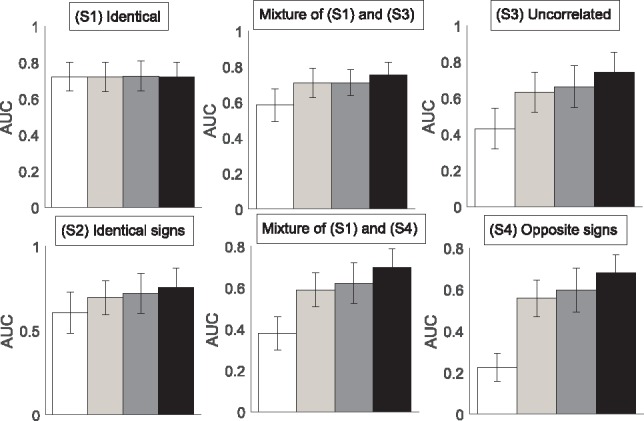
Network reconstruction accuracy for RAF pathway. The histograms show the average precision-recall AUC values. Each AUC is averaged across 25 datasets and the error bars indicate SDs. The bars refer to: the homogeneous DBN (white), the NH-DBN model (light-grey), the coupled NH-DBN (dark-grey) and the partially NH-DBN (black). For (S2–5) the AUC differences are significantly in favour of the new partially NH-DBN (two-sided paired *t*-test *P*-values < 0.05)

For each scenario we generate 25 datasets. We then analyse every dataset with each model. Figure 3 shows the average AUC values for reconstructing the RAF pathway. Only for scenario (S1), where all coefficients are constant, the models perform equally well. For (S2–S6) the homogeneous DBN is substantially worse than the NH-DBNs. The coupled NH-DBN is slightly superior to the (non-coupled) NH-DBN. The proposed partially NH-DBN yields the highest average AUC scores.

### 4.3 Pre-study 3: Scalability and computational costs

To study the scalability of the new network reconstruction method, we generate data for random network structures with N∈{10,25,50,100} nodes. For each node *Z_i_* we first sample the number of parents *x_i_* from a Poisson distribution with parameter λ = 1 (‘Poisson in-degree distribution’), before we randomly draw a parent set $\Pi_{i}$ from a uniform distribution over the system of all parent sets with cardinality *x_i_*, $\left\{\Pi_{i}:|\Pi_{i}|=x_{i}\right\}$. Given the network structure, we generate data as described in Section 4.2, i.e. via regression relationships using *K* = 2 and *T_k_* = 10. Here we present and discuss the results for the scenario: ‘Mixture of (S1) and (S3)’. For each *N* we generate 10 independent datasets, i.e. 40 in total. Next, we measure how many MCMC iterations *W* we can perform in 1 h. With our Matlab implementation on a desktop computer with Intel Xeon 2.5 GHz processor and 8GB of RAM, the average numbers of iterations per hour are: *W* = 208 637 (*N* = 10), *W* = 83 615 (*N* = 25), *W* = 41 666 (*N* = 50) and *W* = 19 855 (*N* = 100).

To monitor convergence and network reconstruction accuracy in real-time, we perform long MCMC simulations. For each *N* we select the numbers of iterations such that the simulation takes 16 h. During the simulations we sample 200 equidistant networks per hour (i.e. 3200 networks in total). When withdrawing the first 50% of the networks (‘burn-in’), we have $R_{h}=100h$ networks after *h* hours. We use those *R_h_* networks to assess the performance after *h* hours of computational time. For running two independent 16 h long MCMC simulations on each of the 40 datasets (computational time: 1280 h), we use a computer cluster.

**Fig. 4.**
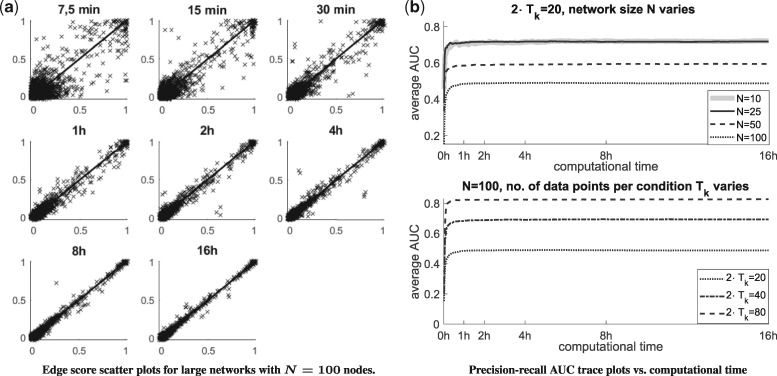
Convergence analysis. **(a)** For each of 10 datasets two independent MCMC simulations have been performed. The edge scores of the two simulations can be plotted against each other. Each panels refer to a computational time and show 10 superimposed scatter plots. **(b)** Both panels show curves of the average AUC value against the computational time. In the upper panel the network size *N* varies, while the no. of data points is kept fixed. In the lower panel for *N* = 100 the no. of data points is varied

To assess convergence, we consider scatter plots of the edge scores of two independent MCMC simulations on the same dataset. For the largest networks with *N* = 100, Figure 4a shows superimposed scatter plots for different computational times. It can be seen that after 2–4 h only few edges points deviate from the diagonal, i.e. only few edge scores differ between independent simulations. This is a good indication of convergence. The corresponding scatter plots for the smaller networks with N∈{10,25,50} can be found in Part F of the Supplementary Material. As expected, the Supplementary Figures show that the rate of convergence decreases with the size of the network *N*.

The upper panel of Figure 4b monitors the average precision-recall AUC scores along the computational time. The four AUC curves run into plateaus. Already after 1–2 h the AUCs (i.e. the average network reconstruction accuracy) does not improve anymore. The two curves for *N* = 10 and = 25 converge to AUC = 0.72, while the AUC limits are lower for *N* = 50 (AUC = 0.58) and *N* = 100 (AUC = 0.49). The individual AUC curves are shown in Part F of the Supplementary Material. Taking into account that a network among *N* = 100 nodes had to be inferred from 20 data points (*K* = 2 conditions with *T_k_* = 10 data points each), the rather low network reconstruction accuracy (AUC = 0.49) is not surprising. To show that higher AUCs can be reached for networks with *N* = 100 nodes, we repeat the study with *T_k_* = 20 and = 40. The bottom panel of Figure 4b monitors the average AUC scores for *N* = 100 and $T_{k}\in \{10,20,40\}$. Here, we had to adapt the numbers of MCMC iterations per hour to *W* = 12 707 (*T_k_* = 20), and *W* = 9343 ($T_{k}=40)$. The new curves also run into plateaus and reach higher limits: AUC = 0.70 and = 0.83. The results of this section are compactly summarized in Table 1.

**Table 1.** Summary of scalability results

Nodes *N*	10	25	50	100	100	100
Data points $2\cdot T_{k}$	20	20	20	20	40	80
Iterations per hour	208 637	83 615	41 666	19 855	12 707	9 343
AUC limit	0.72	0.72	0.58	0.49	0.70	0.83

*Note*: See Section 4.3 for further details.

### 4.4 Reconstructing the yeast gene network topology

**Fig. 5.**
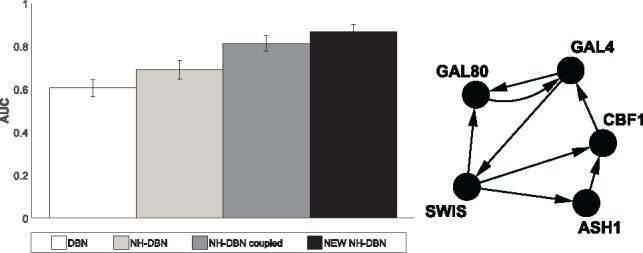
Network reconstruction accuracy for yeast gene expression data. The histogram shows the average precision-recall AUC values, averaged across 25 MCMC simulations, with error bars indicating SDs. The AUCs are: 0.61 (DBN), 0.69 (NH-DBN), 0.81 (coupled NH-DBN) and 0.87 (new NH-DBN). All three AUC differences are significant in terms of two-sided *t*-tests (*P* < 10^–3^)

By means of synthetic biology, Cantone *et al.* (2009) designed a network with *N* = 5 genes in *S.cerevisiae* (yeast); Figure 5 shows the true network. With quantitative Real-Time Polymerase Chain Reaction, Cantone *et al.* (2009) then measured *in vivo* gene expression data: under galactose- (*k* = 1) and glucose-metabolism (*k* = 2). $T_{1}=16$ measurements were taken in galactose and $T_{2}=21$ in glucose. The data have become a benchmark application, as the network reconstruction accuracies can be cross-compared on real *in vivo* gene expression data. Figure 5 shows the results, and again a clear trend can be seen: The homogeneous DBN yields the lowest AUC value. The NH-DBN model yields higher AUCs and can be further improved by coupling the regression coefficients (coupled NH-DBN). The proposed partially NH-DBN reaches the highest network reconstruction accuracy. The results are thus consistent with the results for the RAF-pathway data in Section 4.2.

### 4.5 The circadian clock network in *A.thaliana*

**Fig. 6.**
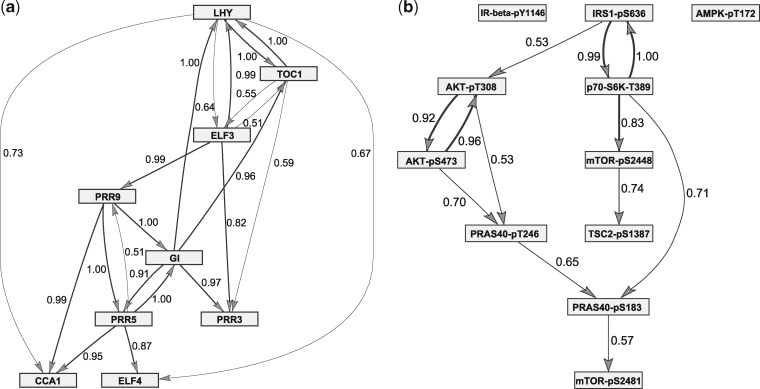
Shown are the edges whose scores exceeded the threshold ψ=0.5; edges are labelled with their scores. Edges with scores higher than ψ=0.8 are in bold. (**a**) Inferred circadian clock network in *Arabidopsis thaliana*. (**b**) Inferred topology of the mTORC1 signalling pathway

The circadian clock network in *A.thaliana* orchestrates the gene regulatory processes, related to the plant metabolism, with respect to the daily changing dark: light cycles of the solar day. The mechanism of internal time-keeping allows the plant to anticipate each new day, at the molecular level, and to optimize its growth. In *K* = 4 experiments *Arabidopsis* plants were entrained in different dark: light cycles, before the gene expressions of *N* = 9 circadian clock genes were measured under experimentally controlled constant light condition. The numbers of observed time points are $T_{1}=12$ and *T_k_* = 13 for *k* = 2, 3, 4. For further details on the experimental protocols we refer to Grzegorczyk (2016). Figure 6a shows a network that was inferred with the new partially coupled model. For the prediction, we extracted the 21 edges with edge scores higher than the threshold ψ=0.5. A proper biological evaluation of the network is hindered and beyond the scope of this article, as the true circadian clock network has not been fully discovered yet. However, our predicted network in Figure 6a contains many edges that are consistent with hypotheses from the plant biology literature. In particular, the high-scoring feedback loop LHY ↔TOC1 seems to be the most important key feature of the circadian clock network (see, e.g. Locke *et al.*, 2006). Moreover, it has been reported that LHY is a regulator of the genes ELF3 and ELF4 (Alabadi *et al.*, 2001; Kikis *et al.*, 2005). Also the edge LHY →CCA1 is not unexpected, as LHY and CCA1 are known to be biological homologues (Miwa *et al.*, 2007). Four more edges, for which we could find biological literature references, are: GI →TOC1 (Locke *et al.*, 2005), ELF3 →LHY (Kikis *et al.*, 2005), ELF3 →TOC1 (Dixon *et al.*, 2011) and ELF3 →PRR9 (Chow *et al.*, 2012).

### 4.6 The mTORC1 network

The mammalian target of rapamycin complex 1 (mTORC1) is a serine/threonine kinase which is evolutionary conserved and essential in all eukaryotes (Saxton and Sabatini, 2017). mTORC1 is at the centre of a multiply wired, complex signalling network, whose topology is well studied and contains several well-characterized feedback loops (Saxton and Sabatini, 2017). Hence, we used the mTORC1 network as a surrogate based on which we can objectively evaluate the predictive power of our partially NH-DBN model for learning network structures. The signalling network converging on mTORC1 is built by kinases, which inactivate or activate each other by phosphorylation. Thus, a protein can be phosphorylated at one or several sites, and the phosphorylations at these positions determine its activity. Signalling through the mTORC1 network is elicited by external signals like insulin or amino acids. Dalle Pezze *et al.* (2016) relatively quantified 11 phosphorylation states of 8 key proteins across the mTORC1 signalling network by immunoblotting; for an overview see Table 2. Dynamic time course data were obtained under two experimental conditions, namely upon stimulation with amino acids only (*k* = 1), and with amino acids plus insulin (*k* = 2). The phosphorylation states were measured at *T_k_* = 10 time points: t=0,1,3,5,10,15,30,45,60,120 minutes, so that the time lag increases from 1 to 60. We therefore apply the GP method from Section 2.5 to predict equidistant response values, before analysing the data with the proposed partially NH-DBN. The 12 edges with scores higher than ψ=0.5 yield the network topology shown in Figure 6b. A literature review shows that 11 out of the 12 edges have been reported earlier.

**Table 2.** mTORC1 timecourse data

Protein	Full name	Sites
mTOR	mammalian target of rapamycin	pS2481, pS2448
PRAS40	proline-rich AKT/PKB substrate 40 kDa	pT246, pS183
AKT	Protein kinase B	pT308, pS473
IRS1	insulin receptor substrate 1	pS636
IR-beta	insulin receptor beta	pY1146
AMPK	AMP-dependent protein kinase	pT172
TSC2	tuberous sclerosis 2 protein	pS1387
p70-S6K	Ribosomal protein S6 kinase beta-1	pT389

*Note*: Overview to the 8 proteins and the 11 measured phosphorylation sites.

We focus first on the five interactions with the highest scores ψ>0.8. Two out of these five interactions are enzyme-substrate relationships: p70-S6K is a kinase which is directly activated by mTORC1 through phosphorylation at threonine 389 [p70-S6K-pT389] (Saxton and Sabatini, 2017). Thus, p70-S6K-pT389 represents a direct readout of mTORC1 activity. p70-S6K phosphorylates IRS1 at serine 636, [IRS1-pS636] (Tzatsos and Kandor, 2006) and mTOR at serine 2448 [mTOR-pS2448] (Dibble and Cantley, 2015), and both edges are correctly identified by our model [p70-S6K-pT389→IRS1-pS636, p70-S6K-pT389→mTOR-pS2448]. Two other interactions with a high score are between AKT-pT308 ↔AKT-pS473. The two phosphorylations are predicted by our model to influence each other, and a positive feedback between phosphorylation events on S473 and T308 of AKT has indeed been demonstrated biochemically (Manning and Toker, 2017). Another high score prediction is between IRS1-pS636 and p70-S6K-pT389 [IRS1-pS636 →p70-S6K-pT389]. Phosphorylation at S636 inhibits IRS1, thereby leading to inhibition of mTORC1 and its substrate p70-S6K-T389 (Tzatsos and Kandor, 2006). Thus, the negative feedback between IRS1-pS639 and p70-S6K-pT389 explains the learned edge between them [IRS1-pS636→p70-S6K-pT389]. In addition, IRS1 inhibition by phosphorylation at S636 results in reduced phosphorylation of AKT at threonine 308, which is in agreement with the learned edge between IRS1-pS636 and AKT-pT308 [IRS1-pS636→AKT-pT308].

We could also find evidence for 6 of the remaining 7 edges with scores in between 0.5 and 0.8. PRAS40 is an endogenous mTORC1 inhibitor (Saxton and Sabatini, 2017). The edge from PRAS40-pT246 to PRAS40-pS183 corresponds to a well-described mechanism of PRAS40 regulation: AKT phosphorylates PRAS40 at T246 [PRAS40-pT246], which allows subsequent phosphorylation of PRAS40-S183 by mTORC1 (Nascimento *et al.*, 2010). This interaction is accurately resembled by our model [PRAS40-pT246→ PRAS40-pS183]. PRAS40’s double phosphorylation dissociates PRAS40 from mTORC1, leading to its derepression (Nascimento *et al.*, 2010). This mechanism is resembled by the edge between PRAS40-S183 and mTOR-S2481 [PRAS40-pS183→mTOR-pS2481], the latter being an autophosphorylation site which directly monitors mTOR activity (Soliman *et al.*, 2010). Furthermore, the model suggests an edge between p70-S6K-pT389 and PRAS40-pS183 [p70-S6K-pT389 →PRAS40-pS183]. Both are mTORC1 substrate sites (Nascimento *et al.*, 2010; Saxton and Sabatini, 2017) and are therefore often targeted in parallel. The only predicted edge for which there is to the best of our knowledge no literature evidence is between mTOR-pS2448 and TSC2-pS1387 [mTOR-pS2448 →TSC2-pS1387]. TSC2 is activated by phosphorylation at S1387 and inhibits mTORC1 (Hindupur *et al.*, 2015). Our model prediction that mTORC1—when phosphorylated at S2448 by p70-S6K—regulates TSC2 remains to be experimentally tested.

After having identified 11 of 12 edges as true positives, we performed a literature review to find false negative edges, i.e. edges that our model did not extract, although it has been reported that they exist. This way, we could identify two false negative edges, namely: IR-beta-pY1146→AKT-pT308 and AMPK-pT172→TSC2-pS1387, which were reported in Vigneri *et al.* (2016) and Mihaylova and Shaw (2012), respectively. Finally, we note that this does not imply that the remaining edges that our model did not extract can be assumed to be true negatives. Nowadays incomplete knowledge about the topology of the mTORC1 pathway renders the safe identification of true negative edges impossible. The absence of literature reports on an edge does not necessarily imply that it does not exist.

## 5 Conclusion and discussion

We propose a new partially NH-DBN model for learning network structures. When data are measured under different experimental conditions, it is rarely clear whether the data can be merged and analysed within one single model, or whether there is need for a NH-DBN model that allows the network parameters to depend on the condition. The new partially NH-DBN has been designed such that it can infer the best trade-off from the data. It infers for each individual edge whether the corresponding interaction parameter is constant or condition-specific. Our applications to synthetic RAF pathway data as well as to yeast gene-expression data have shown that the partially NH-DBN model improves the network reconstruction accuracy. We have used the partially NH-DBN model to predict the structure of the mTORC1 signalling network. As the measured mTORC1 data are non-equidistant, we have applied a GP-based method to predict the missing equidistant values. Results on synthetic data (see Section 4.1) show that the proposed GP-method (see Section 2.5) can lead to substantially improved results.

All but one of the predicted interactions across the mTORC1 network are reflected in experiments reported in the biological literature. Dalle Pezze *et al.* (2016) built an ODE-based dynamic model which allows to predict signalling responses to perturbations. Like for many ODE-based models, the topology of this model was defined by the authors, based on literature-knowledge. The ODE model simulations could reproduce the measured mTORC1 timecourse data. Interestingly, all the connections predicted by our new partially NH-DBN model form part of the core model by Dalle Pezze *et al.* (2016). Hence, we present an alternative unsupervised learning approach, in which the topology of signalling networks is inferred directly from the data. The new model is thus a complementary tool that enhances dynamic model building by predicting the network’s topology in a purely data-driven manner.

Although it worked well for the mTORC1 data, we note that the GP method from Section 2.5 requires the time series to be sufficiently smooth. For non-smooth time series the method might not be able to properly predict the values at unobserved time points, leading to biased response values. Then, network reconstruction methods, such as the new partially NH-DBN, will inevitably infer distorted network topologies and wrong conclusions might be drawn. The assumption of smoothness is therefore crucial for the complete analysis pipeline to work. Our results in Section 4.3 show that the new partially NH-DBN can also be used to infer larger networks. However, our results suggest that there is then need for more data points (or higher signal-to-noise ratios, respectively) to reach accurate network predictions. A conceptual advantage of our partially NH-DBN is that it has two established models, namely the homogeneous DBN (δ=1) and the globally coupled NH-DBN (δ=0) as limiting cases. The new model operates between them, and as we follow a model averaging approach, it is less susceptible to over-fitting. The edge scores of the partially NH-DBN take the two established models as well as all ‘in-between’ models into account. For sparse data, we would thus expect low edge scores, indicating that we might average across too many models.

## Funding

M.S.K. and M.G. are supported by the European Cooperation in Science and Technology (COST) [COST Action CA15109 European Cooperation for Statistics of Network Data Science (COSTNET)]. K.T. acknowledges support from the BMBF e: Med initiatives GlioPATH [01ZX1402B] and MAPTor-NET (031A426B), the German Research Foundation [TH 1358/3-1], the Stichting TSC Fonds (calls 2015 and 2017) and the MESI-STRAT project, which has received funding from the European Union’s Horizon 2020 research and innovation programme under [grant agreement No 754688]. K.T. is recipient of a Rosalind-Franklin-Fellowship of the University of Groningen and of the Research Award 2017 of the German Tuberous Sclerosis Foundation.

*Conflict of Interest*: none declared.

## Supplementary Material

bty917_Supplementary_Data

## References

[bty917-B1] Aderhold A. et al (2014) Statistical inference of regulatory networks for circadian regulation. Stat. Appl. Genet. Mol. Biol., 13, 227–273. 24864301 10.1515/sagmb-2013-0051

[bty917-B2] Ahmed A., Xing E. (2009) Recovering time-varying networks of dependencies in social and biological studies. Proc. Natl. Acad. Sci. USA, 106, 11878–11883. 19570995 10.1073/pnas.0901910106 PMC2704856

[bty917-B3] Alabadi D. et al (2001) Reciprocal regulation between TOC1 and LHY/CCA1 within the Arabidopsis circadian clock. Science, 293, 880–883. 11486091 10.1126/science.1061320

[bty917-B4] Bishop C.M. (2006). Pattern Recognition and Machine Learning. Springer, Singapore.

[bty917-B5] Cantone I. et al (2009) A yeast synthetic network for in vivo assessment of reverse-engineering and modeling approaches. Cell, 137, 172–181. 19327819 10.1016/j.cell.2009.01.055

[bty917-B6] Chow B. et al (2012) ELF3 recruitment to the PRR9 promoter requires other evening complex members in the Arabidopsis circadian clock. Plant Signal Behav., 7, 170–173. 22307044 10.4161/psb.18766 PMC3405715

[bty917-B7] Dalle Pezze P. et al (2016) A systems study reveals concurrent activation of AMPK and mTOR by amino acids. Nat. Commun., 7, 1–19. 10.1038/ncomms13254 PMC5121333 27869123

[bty917-B8] Davis J., Goadrich M. (2006). The relationship between precision-recall and ROC curves In: ICML ’06: Proceedings of the 23rd International Conference on Machine Learning, pages 233–240. ACM, New York, NY, USA.

[bty917-B9] Dibble C., Cantley L. (2015) Regulation of mTORC1 by PIP3K signaling. Trends Cell Biol., 25, 545–555. 26159692 10.1016/j.tcb.2015.06.002 PMC4734635

[bty917-B10] Dixon L. et al (2011) Temporal repression of core circadian genes is mediated through EARLY FLOWERING 3 in Arabidopsis. Curr. Biol., 21, 120–125. 21236675 10.1016/j.cub.2010.12.013 PMC3028277

[bty917-B11] Dondelinger F. et al (2013) Non-homogeneous dynamic Bayesian networks with Bayesian regularization for inferring gene regulatory networks with gradually time-varying structure. Mach. Learn., 90, 191–230.

[bty917-B12] Friedman N. et al (2000) Using Bayesian networks to analyze expression data. J. Comput. Biol., 7, 601–620. 11108481 10.1089/106652700750050961

[bty917-B13] Geissen E. et al (2016) MEMO: multi-experiment mixture model analysis of censored data. Bioinformatics, 32, 2464–2472. 27153627 10.1093/bioinformatics/btw190 PMC4978932

[bty917-B14] Green P. (1995) Reversible jump Markov chain Monte Carlo computation and Bayesian model determination. Biometrika, 82, 711–732.

[bty917-B15] Grzegorczyk M. (2016) A non-homogeneous dynamic Bayesian network with a hidden Markov model dependency structure among the temporal data points. Mach. Learn., 102, 155–207.

[bty917-B16] Grzegorczyk M., Husmeier D. (2013) Regularization of non-homogeneous dynamic Bayesian networks with global information-coupling based on hierarchical Bayesian models. Mach. Learn., 91, 105–154.

[bty917-B17] Hindupur S. et al (2015) The opposing actions of target of rapamycin and AMP-activated protein kinase in cell growth control. Cold Spring Harb. Perspect. Biol., 7, a019141. 26238356 10.1101/cshperspect.a019141 PMC4526743

[bty917-B18] Husmeier D. et al (2010). Inter-time segment information sharing for non-homogeneous dynamic Bayesian networks In: Lafferty J. E. A. (ed.) Proceedings of the Twenty-Fourth Annual Conference on Neural Information Processing Systems (NIPS), Vol. 23, pp. 901–909. Curran Associates.

[bty917-B19] Kikis E. et al (2005) ELF4 is a phytochrome-regulated component of a negative-feedback loop involving the central oscillator components CCA1 and LHY. Plant J., 44, 300–313. 16212608 10.1111/j.1365-313X.2005.02531.x

[bty917-B20] Lèbre S. et al (2010) Statistical inference of the time-varying structure of gene-regulation networks. BMC Syst. Biol., 4, 10.1186/1752-0509-4-130 PMC2955603 20860793

[bty917-B21] Locke J. et al (2005) Extension of a genetic network model by iterative experimentation and mathematical analysis. Mol. Syst. Biol., 1, 13. Doi: 10.1038/msb4100018. 10.1038/msb4100018 PMC1681447 16729048

[bty917-B22] Locke J.C.W. et al (2006) Experimental validation of a predicted feedback loop in the multi-oscillator clock of Arabidopsis thaliana. Mol. Syst. Biol., 2, 59. Doi: 10.1038/msb4100102. 10.1038/msb4100102 PMC1682024 17102804

[bty917-B23] Manning B., Toker A. (2017) AKT/PKB Signaling: navigating the Network. Cell, 169, 381–405. 28431241 10.1016/j.cell.2017.04.001 PMC5546324

[bty917-B24] Mihaylova M.M., Shaw R. (2012) The AMP-activated protein kinase (AMPK) signaling pathway coordinates cell growth, autophagy, & metabolism. Nat. Cell Biol., 13, 1016–1023. 10.1038/ncb2329 PMC3249400 21892142

[bty917-B25] Miwa K. et al (2007) Genetic linkages of the circadian clock-associated genes, TOC1, CCA1 and LHY, in the photoperiodic control of flowering time in Arabidopsis thaliana. Plant Cell Physiol., 48, 925–937. 17540692 10.1093/pcp/pcm067

[bty917-B26] Nascimento E. et al (2010) Phosphorylation of PRAS40 on Thr246 by PBK/AKT facilitates efficient phosphorylation of Ser183 by mTORC1. Cell. Signal., 22, 961–967. 20138985 10.1016/j.cellsig.2010.02.002

[bty917-B27] Robinson J., Hartemink A. (2010) Learning non-stationary dynamic Bayesian networks. J. Mach. Learn. Res., 11, 3647–3680.

[bty917-B28] Sachs K. et al (2005) Causal protein-signaling networks derived from multiparameter single-cell data. Science, 308, 523–529. 15845847 10.1126/science.1105809

[bty917-B29] Saxton R., Sabatini D. (2017) mTOR signaling in growth, metabolism, and disease. Cell, 168, 960–976. 28283069 10.1016/j.cell.2017.02.004 PMC5394987

[bty917-B30] Soliman G. et al (2010) mTOR Ser-2481 autophosphorylatyion monitors mTORC-specific catalytic activity and clarifies rapamycin mechanism of action. J. Biol. Chem., 285, 7866–7879. 20022946 10.1074/jbc.M109.096222 PMC2832937

[bty917-B31] Tzatsos A., Kandror K.V. (2006) Nutrients suppress phosphatidylinositol 3-kinase/AKT signaling via raptor-dependent mTOR-mediated insulin receptor substrate 1 phosphorylation. Mol. Cell Biol., 26, 63–76. 16354680 10.1128/MCB.26.1.63-76.2006 PMC1317643

[bty917-B32] Vanhatalo J. et al (2013) GPstuff: Bayesian modeling with Gaussian processes. J. Mach. Learn. Res., 14, 1175–1179.

[bty917-B33] Vigneri R. et al (2016) Insulin, insulin receptors, and cancer. J. Endocrinol. Investig., 39, 1365–1376. 27368923 10.1007/s40618-016-0508-7

