# Supplementary material for: Partially non-homogeneous dynamic Bayesian networks based on Bayesian regression models with partitioned design matrices
Source: Bioinformatics. 2018 Nov 5;35(12):2108–17. doi: 10.1093/bioinformatics/bty917 (PMC6581439; doi:10.1093/bioinformatics/bty917)
Supplement: bty917_Supplementary_Data [file bty917_supplementary_data.pdf]

Supplementary material
for our paper:
'Partially non-homogeneous dynamic Bayesian networks
based on
Bayesian regression models with partitioned design matrices'
(submitted to Bioinformatics)

M. Shafiee Kamalabad, A.M. Heberle, K. Thedieck and M. Grzegorczyk

Part 0 - Summary from the main paper

For the posterior of the proposed partially NH-DBN model we have:

$$p(\boldsymbol{\beta}_B, \sigma^2, \lambda_\star^2, \lambda_\diamond^2, \boldsymbol{\mu}|\mathbf{y}) \propto p(\mathbf{y}|\sigma^2, \boldsymbol{\beta}_B) \cdot p(\boldsymbol{\beta}_B|\sigma^2, \lambda_\diamond^2, \lambda_\star^2, \boldsymbol{\mu}) \cdot p(\boldsymbol{\mu}) \cdot p(\sigma^{-2}) \cdot p(\lambda_\star^{-2}) \cdot p(\lambda_\diamond^{-2}) \quad (1)$$

and the model likelihood is given by:

$$\mathbf{y} \sim \mathcal{N}_T(\mathbf{X}_B \boldsymbol{\beta}_B, \sigma^2 \mathbf{I})$$

where $\boldsymbol{\beta}_B$ is a vector of $(1 + n_1 + K \cdot n_2)$ regression coefficients, and $\mathbf{X}_B$ is a partitioned matrix with $\sum(T_k - 1)$ rows and $(1 + n_1) + (K \cdot n_2)$ columns. E.g. for $K = 2$, when the intercept and the first $n_1 < n$ coefficients stay constant while the remaining $n_2 = n - n_1$ coefficients are component-specific, the matrix $\mathbf{X}_B$ has the structure:

$$\mathbf{X}_B = \begin{pmatrix} \mathbf{1} & \mathbf{x}_{1,1} & \dots & \mathbf{x}_{n_1,1} & \mathbf{x}_{n_1+1,1} & \dots & \mathbf{x}_{n,1} & \mathbf{0} & \dots & \mathbf{0} \\ \mathbf{1} & \mathbf{x}_{1,2} & \dots & \mathbf{x}_{n_1,2} & \mathbf{0} & \dots & \mathbf{0} & \mathbf{x}_{n_1+1,2} & \dots & \mathbf{x}_{n,2} \end{pmatrix},$$

In the main paper we have shown that $\boldsymbol{\beta}_B$ has a Gaussian prior:

$$\boldsymbol{\beta}_B|(\sigma^2, \lambda_\diamond^2, \lambda_\star^2, \boldsymbol{\mu}) \sim \mathcal{N}_{1+n_1+K\cdot n_2}(\tilde{\boldsymbol{\mu}}, \sigma^2 \tilde{\boldsymbol{\Sigma}}) \quad \text{with: } \tilde{\boldsymbol{\mu}} = \begin{pmatrix} \mathbf{0} \\ \boldsymbol{\mu} \\ \vdots \\ \boldsymbol{\mu} \end{pmatrix} \quad \text{and } \tilde{\boldsymbol{\Sigma}} = \begin{pmatrix} \lambda_\star^2 \mathbf{I}_\star & \mathbf{0} \\ \mathbf{0} & \lambda_\diamond^2 \mathbf{I}_\diamond \end{pmatrix}$$

where $\mathbf{I}_\star$ is the $(n_1 + 1)$-dimensional identity matrix, and $\mathbf{I}_\diamond$ is the $(K \cdot n_2)$-dimensional identity matrix. Moreover, we have imposed the following priors:

$$\sigma^{-2} \sim \text{GAM}(a, b) \text{ and } \lambda_\star^{-2} \sim \text{GAM}(\alpha_\star, \beta_\star) \text{ and } \lambda_\diamond^{-2} \sim \text{GAM}(\alpha_\diamond, \beta_\diamond) \text{ and } \boldsymbol{\mu} \sim \mathcal{N}_{n_2}(\boldsymbol{\mu}_0, \boldsymbol{\Sigma}_0)$$

When deriving the full conditional distributions we will use the relationship:

$$\mathbf{X}_B \cdot \tilde{\boldsymbol{\mu}} = \mathbf{X}_B^\ddagger \cdot \boldsymbol{\mu} \quad \text{where} = \quad \mathbf{X}_B^\ddagger := \begin{pmatrix} \mathbf{x}_{n_1+1,1} & \dots & \mathbf{x}_{n,1} \\ \mathbf{x}_{n_1+1,2} & \dots & \mathbf{x}_{n,2} \\ \vdots & \dots & \vdots \\ \mathbf{x}_{n_1+1,K} & \dots & \mathbf{x}_{n,K} \end{pmatrix} \quad (2)$$

Part A - Deriving the full conditional distributions

A sample from the posterior distribution in Equation (1) can be generated by Markov Chain Monte Carlo (MCMC) simulations. In this subsection we derive the full conditional distributions (FCDs) for the model parameters: $\boldsymbol{\beta}_B$, $\lambda_\star^2$, and $\lambda_\diamond^2$. For σ^2 and $\boldsymbol{\mu}$ we implement collapsed Gibbs sampling moves. In collapsed Gibbs sampling steps some of the other variables are integrated out analytically from the FCDs. Collapsed Gibbs sampling steps are known to be more efficient than standard Gibbs sampling steps. Within a Gibbs MCMC sampling scheme all parameters are iteratively resampled from their FCDs or by a collapsed Gibbs sampling step.

The densities of the FCDs are proportional to the factorized joint density in Equation (1). From the shape of the densities we conclude what the full conditional distributions (FCDs) are.

For the **full conditional distribution** of $\boldsymbol{\beta}_B$ we obtain:

$$\begin{aligned}
\text{FCD}(\boldsymbol{\beta}_B) &\propto p(\mathbf{y}|\sigma^2,\boldsymbol{\beta}_B)\cdot p(\boldsymbol{\beta}_B|\sigma^2,\lambda_\diamond^2,\lambda_\star^2,\boldsymbol{\mu}) \\
&\propto \exp\{-\frac{1}{2}(\mathbf{y}-\mathbf{X}_B\boldsymbol{\beta}_B)^\mathsf{T}(\sigma^2\mathbf{I})^{-1}(\mathbf{y}-\mathbf{X}_B\boldsymbol{\beta}_B)\}\cdot\exp\{-\frac{1}{2}(\boldsymbol{\beta}_B-\tilde{\boldsymbol{\mu}})^\mathsf{T}(\sigma^2\tilde{\boldsymbol{\Sigma}})^{-1}(\boldsymbol{\beta}_B-\tilde{\boldsymbol{\mu}})\} \\
&\propto \exp\{-\frac{1}{2}\cdot\boldsymbol{\beta}_B^\mathsf{T}\left(\sigma^{-2}[\tilde{\boldsymbol{\Sigma}}^{-1}+\mathbf{X}_B^\mathsf{T}\mathbf{X}_B]\right)\boldsymbol{\beta}_B \;+\; \boldsymbol{\beta}_B^\mathsf{T}\left(\sigma^{-2}(\tilde{\boldsymbol{\Sigma}}^{-1}\tilde{\boldsymbol{\mu}}+\mathbf{X}_B^\mathsf{T}\mathbf{y})\right)\}
\end{aligned}$$

and from the shape of the latter density we conclude:

$$\boldsymbol{\beta}_B|(\sigma^2,\lambda_\diamond^2,\lambda_\star^2,\boldsymbol{\mu})\sim\mathcal{N}_{1+n_1+K\cdot n_2}\left([\tilde{\boldsymbol{\Sigma}}^{-1}+\mathbf{X}_B^\mathsf{T}\mathbf{X}_B]^{-1}(\tilde{\boldsymbol{\Sigma}}^{-1}\tilde{\boldsymbol{\mu}}+\mathbf{X}_B^\mathsf{T}\mathbf{y})\ ,\ \sigma^2[\tilde{\boldsymbol{\Sigma}}^{-1}+\mathbf{X}_B^\mathsf{T}\mathbf{X}_B]^{-1}\right) \tag{3}$$

For the **full conditional distributions** of $\lambda_\diamond^2$ and $\lambda_\star^2$ we get:

$$\begin{aligned}
\text{FCD}(\lambda_\diamond^2) &\propto p(\lambda_\diamond^{-2})\cdot p(\boldsymbol{\beta}_B|\sigma^2,\lambda_\diamond^2,\lambda_\star^2,\boldsymbol{\mu}) \\
&\propto p(\lambda_\diamond^{-2})\cdot\prod_{k=1}^{K}p(\boldsymbol{\beta}_k|\sigma^2,\lambda_\diamond^2) \\
&\propto (\lambda_\diamond^{-2})^{\alpha_\diamond+\frac{Kn_2}{2}-1}\cdot\exp\{-\lambda_\diamond^{-2}(\beta_\diamond+\frac{1}{2}\sigma^{-2}\sum_{k=1}^{K}(\boldsymbol{\beta}_k-\boldsymbol{\mu})^\mathsf{T}(\boldsymbol{\beta}_k-\boldsymbol{\mu}))\}
\end{aligned}$$

$$\begin{aligned}
\text{FCD}(\lambda_\star^2) &\propto p(\lambda_\star^{-2})\cdot p(\boldsymbol{\beta}_B|\sigma^2,\lambda_\diamond^2,\lambda_\star^2,\boldsymbol{\mu}) \\
&\propto p(\lambda_\star^{-2})\cdot p(\boldsymbol{\beta}_\star|\sigma^2,\lambda_\star^2) \\
&\propto (\lambda_\star^{-2})^{\alpha_\star+\frac{n_1}{2}-1}\cdot\exp\{-\lambda_\star^{-2}(\beta_\star+\frac{1}{2}\sigma^{-2}\boldsymbol{\beta}_\star^\mathsf{T}\boldsymbol{\beta}_\star)\}
\end{aligned}$$

and from the shapes of the densities it follows for the FCDs:

$$\begin{aligned}
\lambda_\diamond^{-2}|(\sigma^2,\boldsymbol{\beta}_B,\lambda_\star^2,\boldsymbol{\mu}) &\sim GAM\left(\alpha_\diamond+\frac{Kn_2}{2},\beta_\diamond+\frac{1}{2}\sigma^{-2}\sum_{k=1}^{K}(\boldsymbol{\beta}_k-\boldsymbol{\mu})^\mathsf{T}(\boldsymbol{\beta}_k-\boldsymbol{\mu})\right) \\
\lambda_\star^{-2}|(\sigma^2,\boldsymbol{\beta}_B,\lambda_\diamond^2,\boldsymbol{\mu}) &\sim GAM\left(\alpha_\star+\frac{n_1}{2},\beta_\star+\frac{1}{2}\sigma^{-2}\boldsymbol{\beta}_\star^\mathsf{T}\boldsymbol{\beta}_\star\right)
\end{aligned} \tag{4}$$

For the noise variance parameter σ^2 we implement a **collapsed Gibbs sampling step** with $\boldsymbol{\beta}_B$ integrated out. We have:

$$p(\mathbf{y}|\sigma^2,\lambda_\diamond^2,\lambda_\star^2) = \int p(\mathbf{y},\boldsymbol{\beta}_B|\sigma^2,\lambda_\diamond^2,\lambda_\star^2)d\boldsymbol{\beta}_B = \int p(\mathbf{y}|\boldsymbol{\beta}_B,\sigma^2)\cdot p(\boldsymbol{\beta}_B|\sigma^2,\lambda_\diamond^2,\lambda_\star^2)d\boldsymbol{\beta}_B$$

From a standard rule for Gaussian integrals (see, e.g., Section 2.3.2 in Bishop (2006)):

$$\mathbf{y}|\boldsymbol{\beta} \sim \mathcal{N}_T(\mathbf{X}\boldsymbol{\beta},\boldsymbol{\Sigma}) \text{ with } \boldsymbol{\beta} \sim \mathcal{N}_{1+n_1+K\cdot n_2}(\boldsymbol{\mu},\mathbf{S}) \text{ implies } \mathbf{y} \sim \mathcal{N}_T(\mathbf{X}\boldsymbol{\mu},\boldsymbol{\Sigma}+\mathbf{X}\mathbf{S}\mathbf{X}^\mathsf{T})$$

it follows:

$$\mathbf{y}|(\sigma^2,\lambda_\diamond^2,\lambda_\star^2) \sim \mathcal{N}_T(\mathbf{X}_B\tilde{\boldsymbol{\mu}},\sigma^2[\mathbf{I}+\mathbf{X}_B\tilde{\boldsymbol{\Sigma}}\mathbf{X}_B^\mathsf{T}]) \tag{5}$$

This yields:

$$\begin{aligned}
p(\sigma^2|\mathbf{y},\lambda_\diamond^2,\lambda_\star^2,\boldsymbol{\mu}) &\propto p(\mathbf{y}|\sigma^2,\lambda_\diamond^2,\lambda_\star^2)\cdot p(\sigma^{-2})\\
&\propto (\sigma^{-2})^{0.5\sum(T_k-1)}\exp\{-0.5(\mathbf{y}-\mathbf{X}_B\tilde{\boldsymbol{\mu}})^\mathsf{T}\sigma^{-2}[\mathbf{I}+\mathbf{X}_B\tilde{\boldsymbol{\Sigma}}\mathbf{X}_B^\mathsf{T}]^{-1}(\mathbf{y}-\mathbf{X}_B\tilde{\boldsymbol{\mu}})\}\\
&\quad\cdot(\sigma^{-2})^{a-1}\exp\{-b\sigma^{-2}\}
\end{aligned}$$

The shape of the density implies the collapsed Gibbs sampling step:

$$\sigma^{-2}|(\mathbf{y},\lambda_\diamond^2,\lambda_\star^2,\boldsymbol{\mu}) \sim GAM\left(a+\frac{\sum(T_k-1)}{2}, b+\frac{1}{2}(\mathbf{y}-\mathbf{X}_B\tilde{\boldsymbol{\mu}})^\mathsf{T}[\mathbf{I}+\mathbf{X}_B\tilde{\boldsymbol{\Sigma}}\mathbf{X}_B^\mathsf{T}]^{-1}(\mathbf{y}-\mathbf{X}_B\tilde{\boldsymbol{\mu}})\right)$$

For the FCD of $\boldsymbol{\mu}$ we also use a **collapsed Gibbs sampling step** with $\boldsymbol{\beta}_B$ integrated out (cf. Equation (5)), and we use that $\mathbf{X}_B\cdot\tilde{\boldsymbol{\mu}} = \mathbf{X}_B^\ddagger\cdot\boldsymbol{\mu}$ (cf. Equation (2))

$$\begin{aligned}
\text{FCD}(\boldsymbol{\mu}) &\propto p(\mathbf{y}|\sigma^2,\lambda_\diamond^2,\lambda_\star^2)\cdot p(\boldsymbol{\mu})\\
&\propto \exp\{-0.5\sigma^{-2}(\mathbf{y}-\mathbf{X}_B\tilde{\boldsymbol{\mu}})^\mathsf{T}\sigma^{-2}[\mathbf{I}+\mathbf{X}_B\tilde{\boldsymbol{\Sigma}}\mathbf{X}_B^\mathsf{T}]^{-1}(\mathbf{y}-\mathbf{X}_B\tilde{\boldsymbol{\mu}})\}\dots\\
&\quad\dots\cdot\exp\{-0.5(\boldsymbol{\mu}-\boldsymbol{\mu}_0)^\mathsf{T}\boldsymbol{\Sigma}_0^{-1}(\boldsymbol{\mu}-\boldsymbol{\mu}_0)\}\\
&\propto \exp\{-0.5\boldsymbol{\mu}^\mathsf{T}\left((\mathbf{X}_B^\ddagger)^\mathsf{T}(\sigma^{-2}[\mathbf{I}+\mathbf{X}_B\tilde{\boldsymbol{\Sigma}}\mathbf{X}_B^\mathsf{T}]^{-1})\mathbf{X}_B^\ddagger+\boldsymbol{\Sigma}_0^{-1}\right)\boldsymbol{\mu}\dots\\
&\quad\dots+\boldsymbol{\mu}^\mathsf{T}\left[(\mathbf{X}_B^\ddagger)^\mathsf{T}(\sigma^{-2}[\mathbf{I}+\mathbf{X}_B\tilde{\boldsymbol{\Sigma}}\mathbf{X}_B^\mathsf{T}]^{-1})\mathbf{y}+\boldsymbol{\Sigma}_0^{-1}\boldsymbol{\mu}_0\right]\}
\end{aligned}$$

The latter density is proportional to the density of a Gaussian, so that it follows for the FCD:

$$\boldsymbol{\mu}|(\lambda_\diamond^2,\lambda_\star^2,\sigma^2) \sim \mathcal{N}_{n_2}(\boldsymbol{\mu}^\ddagger,\boldsymbol{\Sigma}^\ddagger) \tag{6}$$

where

$$\boldsymbol{\Sigma}^\ddagger = \left((\mathbf{X}_B^\ddagger)^\mathsf{T}\sigma^{-2}[\mathbf{I}+\mathbf{X}_B\tilde{\boldsymbol{\Sigma}}\mathbf{X}_B^\mathsf{T}]^{-1}\mathbf{X}_B^\ddagger+\boldsymbol{\Sigma}_0^{-1}\right)^{-1} \tag{7}$$

$$\boldsymbol{\mu}^\ddagger = \boldsymbol{\Sigma}^\ddagger\left[(\mathbf{X}_B^\ddagger)^\mathsf{T}(\sigma^{-2}[\mathbf{I}+\mathbf{X}_B\tilde{\boldsymbol{\Sigma}}\mathbf{X}_B^\mathsf{T}]^{-1})\mathbf{y}+\boldsymbol{\Sigma}_0^{-1}\boldsymbol{\mu}_0\right] \tag{8}$$

An important model property is that the marginal likelihood, with $\boldsymbol{\beta}_B$ and σ^2 integrated out, can be computed. The marginalization rule from Section 2.3.7 of Bishop (2006) yields:

$$p(\mathbf{y}|\lambda_\diamond^2,\lambda_\star^2,\boldsymbol{\mu}) = \frac{\Gamma(\frac{T}{2}+a)}{\Gamma(a)}\cdot\frac{\pi^{-\frac{T}{2}}(2b)^a}{\det(\mathbf{C})^{1/2}}\left(2b+(\mathbf{y}-\mathbf{X}_B\tilde{\boldsymbol{\mu}})^\mathsf{T}\mathbf{C}^{-1}(\mathbf{y}-\mathbf{X}_B\tilde{\boldsymbol{\mu}})\right)^{-(\frac{T}{2}+a)} \tag{9}$$

where $T := \sum_{k=1}^{K}(T_k-1)$, and $\mathbf{C} := \mathbf{I}+\mathbf{X}_B\tilde{\boldsymbol{\Sigma}}\mathbf{X}_B^\mathsf{T}$.

Part B - Blocked Metropolis Hastings moves for inferring the covariate set and the covariate types

We note that the indicator vector $\boldsymbol{\delta}$ depends on the covariate set $\boldsymbol{\Pi}$, as it contains one indicator variable for each covariate in $\boldsymbol{\Pi}$. Moreover, the expression $\mathbf{X}_B$, $\boldsymbol{\mu}$, $\tilde{\boldsymbol{\Sigma}}$, $\tilde{\boldsymbol{\mu}}$ and $\mathbf{C}$ all depend on both $\boldsymbol{\Pi}$ and $\boldsymbol{\delta}$, though we do not make that explicit in our notation.
As described in the main paper, given the covariate set $\boldsymbol{\Pi}$ and the corresponding indicator vector $\boldsymbol{\delta}$, the partitioned design matrix $\mathbf{X}_B = \mathbf{X}_{B,\boldsymbol{\Pi},\boldsymbol{\delta}}$ can be built, and the marginal likelihood $p(\mathbf{y}|\lambda_\diamond^2, \lambda_\star^2, \boldsymbol{\mu}, \boldsymbol{\Pi}, \boldsymbol{\delta})$ can be computed with Equation (9). Based on the marginal likelihood (with σ^2 and $\boldsymbol{\beta}_B$ integrated out), we obtain as posterior distribution for the extended model:

$$p(\boldsymbol{\Pi}, \boldsymbol{\delta}, \lambda_\star^2, \lambda_\diamond^2, \boldsymbol{\mu}|\mathbf{y}) \quad \propto \quad p(\mathbf{y}|\lambda_\diamond^2, \lambda_\star^2, \boldsymbol{\mu}, \boldsymbol{\Pi}, \boldsymbol{\delta}) \cdot p(\boldsymbol{\Pi}) \cdot p(\boldsymbol{\delta}|\boldsymbol{\Pi}) \cdot p(\boldsymbol{\mu}|\boldsymbol{\Pi}, \boldsymbol{\delta}) \cdot p(\lambda_\star^2) \cdot p(\lambda_\diamond^2) \quad (10)$$

where $p(\boldsymbol{\mu}|\boldsymbol{\Pi}, \boldsymbol{\delta})$ is a Gaussian, whose dimension is the number of component-specific coefficients, p($\boldsymbol{\Pi}$), is a uniform distribution, truncated to the maximal cardinality $|\boldsymbol{\Pi}| \leq 3$, and $p(\boldsymbol{\delta}|\boldsymbol{\Pi})$ has been specified in Section 3.4 of the main paper.

Now it can be seen why the marginal likelihood from Equation (9) is of great importance for model inference. Because of the analytic expression for the marginal likelihood, we can avoid the notorious Reversible Jump Markov Chain Monte Carlo (RJMCMC) issues (Green, 1995). The continuous regression coefficient vector $\boldsymbol{\beta}_B$, whose elements correspond to the covariates in $\boldsymbol{\Pi}$, has been integrated out, and therefore the vector $\boldsymbol{\beta}_B$ does not appear in the posterior distribution in Equation (10). Consequently, the acceptance probabilities of the Metropolis-Hastings criterion can be formulated in terms of the marginal likelihood and thus do not depend on specific $\boldsymbol{\beta}_B$ vectors. When the Metropolis-Hastings acceptance probabilities are based on marginal likelihoods, they always take all possible regression coefficient vector instantiations into account, and there is no need for specific regression coefficient instantiations for newly introduced covariates.
To formulate that differently: In our new partially NH-DBN there is a one-to-one correspondence between the covariates in $\boldsymbol{\Pi}$ and the regression coefficients in $\boldsymbol{\beta}_B$. For a given covariate set $\boldsymbol{\Pi}$, the marginal likelihood value can be thought of as a weighted average across all possible $\boldsymbol{\beta}_B$ vectors for $\boldsymbol{\Pi}$. A change of the discrete covariate set $\boldsymbol{\Pi}$ implies a change of the continuous regression coefficient vector $\boldsymbol{\beta}_B$, but w.r.t. the marginal likelihood there is no need for any specific regression coefficients. The marginal likelihood always takes all possible $\boldsymbol{\beta}_B$ vectors for $\boldsymbol{\Pi}$ into account.
The resulting MCMC algorithm, presented below, is therefore effectively a Reversible Jump Markov Chain Mone Carlo (RJMCMC) scheme (Green, 1995) in the discrete space of covariate sets, where the Jacobian in the Metropolis-Hastings acceptance probabilities is always equal to 1 and can be omitted (Green, 1995).

We use the marginal likelihood from Equation (9) to implement two Metropolis Hastings moves, in which we make use of the concept of blocking (Liang *et al.* (2010)). Blocking is a technique by which variables are not sampled separately, but are merged into blocks that are sampled together. We form two blocks, grouping $\boldsymbol{\delta}$ with $\boldsymbol{\mu}$, and grouping $\boldsymbol{\Pi}$ with $\boldsymbol{\delta}$ and $\boldsymbol{\mu}$. The vector $\boldsymbol{\delta}$ is then always sampled jointly with $\boldsymbol{\mu}$, and the covariate set $\boldsymbol{\Pi}$ is always sampled jointly with $\boldsymbol{\delta}$ and $\boldsymbol{\mu}$.
In Bayesian statistics, the concept of blocking is a widely applied technique to improve convergence of MCMC inference algorithms, see, e.g., Liang *et al.* (2010). Convergence problems often

result from correlations between variables. To resolve this problem, the key idea is to merge correlated variables into blocks and to sample the variables within each block together, rather than to sample them separately.[1]

First Metropolis Hastings move:
Each move on $[\boldsymbol{\delta}, \boldsymbol{\mu}]$ randomly selects one element δ_i of $\boldsymbol{\delta}$ and proposes to switch its value, i.e to replace δ_i by $1-\delta_i$. This yields a new candidate vector $\boldsymbol{\delta}_\bullet$, for which we re-sample $\boldsymbol{\mu}_\bullet$ from its full conditional distribution in Equation (6). The acceptance probability for the move is:

$$A([\boldsymbol{\delta}, \boldsymbol{\mu}] \to [\boldsymbol{\delta}_\bullet, \boldsymbol{\mu}_\bullet]) = \min\left\{1, \frac{p(\mathbf{y}|\lambda_\diamond^2, \lambda_\star^2, \boldsymbol{\mu}_\bullet, \boldsymbol{\Pi}, \boldsymbol{\delta}_\bullet)}{p(\mathbf{y}|\lambda_\diamond^2, \lambda_\star^2, \boldsymbol{\mu}, \boldsymbol{\Pi}, \boldsymbol{\delta})} \cdot \frac{p(\boldsymbol{\delta}_\bullet|\boldsymbol{\Pi})}{p(\boldsymbol{\delta}|\boldsymbol{\Pi})} \cdot \frac{p(\boldsymbol{\mu}_\bullet|\boldsymbol{\Pi}, \boldsymbol{\delta}_\bullet)}{p(\boldsymbol{\mu}|\boldsymbol{\Pi}, \boldsymbol{\delta})} \cdot H\right\} \tag{11}$$

where the Hastings ratio H is equal to the ratio of full conditional densities:

$$H = \frac{p(\boldsymbol{\mu}|\lambda_\diamond^2, \lambda_\star^2, \sigma^2, \boldsymbol{\Pi}, \boldsymbol{\delta})}{p(\boldsymbol{\mu}_\bullet|\lambda_\diamond^2, \lambda_\star^2, \sigma^2, \boldsymbol{\Pi}, \boldsymbol{\delta}_\bullet)}$$

Second Metropolis Hastings move:
For sampling $[\boldsymbol{\Pi}, \boldsymbol{\delta}, \boldsymbol{\mu}]$ we implement 3 moves on the covariate set $\boldsymbol{\Pi}$, which are accompanied by updates of $\boldsymbol{\delta}$ and $\boldsymbol{\mu}$. Each move proposes to replace $[\boldsymbol{\Pi}, \boldsymbol{\delta}, \boldsymbol{\mu}]$ by a new triple $[\boldsymbol{\Pi}_\bullet, \boldsymbol{\delta}_\bullet, \boldsymbol{\mu}_\bullet]$

- In the deletion move (D) we randomly select one covariate $X \in \boldsymbol{\Pi}$, and we propose to remove this covariate from $\boldsymbol{\Pi}$. This yields $\boldsymbol{\Pi}_\bullet$. Removing X makes the corresponding element δ from $\boldsymbol{\delta}$ redundant, so that we remove it as well to obtain $\boldsymbol{\delta}_\bullet$.

- In the addition move (A) we randomly select one covariate $X \notin \boldsymbol{\Pi}$, and we propose to add this covariate to $\boldsymbol{\Pi}$. This yields $\boldsymbol{\Pi}_\bullet$. We flip a coin to to determine the type ($\delta \in \{0,1\}$) of the new covariate. Adding the element δ to $\boldsymbol{\delta}$ yields $\boldsymbol{\delta}_\bullet$.

- In the exchange move (E) we randomly select one covariate $X_\bullet \in \boldsymbol{\Pi}$, and we propose to replace $X_\bullet$ by a randomly selected new covariate $X \notin \boldsymbol{\Pi}$. This yields $\boldsymbol{\Pi}_\bullet$. We then flip a coin to determine the type ($\delta \in \{0,1\}$) of the new covariate. By removing the element $\delta_\bullet$ from $\boldsymbol{\delta}$ and adding the element δ to $\boldsymbol{\delta}$, we obtain $\boldsymbol{\delta}_\bullet$.

Each sub-move (D, A and E) yields a pair $[\boldsymbol{\Pi}_\bullet, \boldsymbol{\delta}_\bullet]$, which we complete to a triple by sampling a new $\boldsymbol{\mu}_\bullet$, conditional on $\boldsymbol{\Pi}_\bullet$ and $\boldsymbol{\delta}_\bullet$, from the full conditional distribution in Equation (6). When randomly selecting the move type (D, A or E) the acceptance probability is:

$$A([\boldsymbol{\Pi}, \boldsymbol{\delta}, \boldsymbol{\mu}], [\boldsymbol{\Pi}_\bullet, \boldsymbol{\delta}_\bullet, \boldsymbol{\mu}_\bullet]) = \min\left\{1, \frac{p(\mathbf{y}|\lambda_\diamond^2, \lambda_\star^2, \boldsymbol{\mu}_\bullet, \boldsymbol{\Pi}_\bullet, \boldsymbol{\delta}_\bullet)}{p(\mathbf{y}|\lambda_\diamond^2, \lambda_\star^2, \boldsymbol{\mu}, \boldsymbol{\Pi}, \boldsymbol{\delta})} \cdot \frac{p(\boldsymbol{\Pi}_\bullet)}{p(\boldsymbol{\Pi})} \cdot \frac{p(\boldsymbol{\delta}_\bullet|\boldsymbol{\Pi}_\bullet)}{p(\boldsymbol{\delta}|\boldsymbol{\Pi})} \cdot \frac{p(\boldsymbol{\mu}_\bullet|\boldsymbol{\Pi}_\bullet, \boldsymbol{\delta}_\bullet)}{p(\boldsymbol{\mu}|\boldsymbol{\Pi}, \boldsymbol{\delta})} \cdot H\right\} \tag{12}$$

[1]To see why 'blocking' is useful, imagine that there are two **correlated** variables A and B with the current values a and b. We further assume that A can only be sampled via a Metropolis-Hastings sampling step, while B can be sampled from its full conditional distribution (i.e. via a Gibbs sampling step). Let's say the model demands a and b to be similar. When the values of A are sampled separately, a newly proposed value for A, say $a_\star$, will have a low acceptance probability if it is no longer similar to the current value b of B. That is, new values for A would be enforced to stay similar to the current value of B, and vice-versa. When blocking both variables, not only the new value for $a_\star$ is proposed, but within the same move also a new value $b_\star$ for B is proposed, where the latter is sampled conditional on the new candidate $a_\star$. The 'blocked' move thus proposes to exchange (a,b) by $(a_\star, b_\star)$. When the candidate $b_\star$ is sampled from its full conditional distribution, conditional on $A = a_\star$, then $b_\star$ is encouraged to be similar to $a_\star$, so that the new state $(a_\star, b_\star)$ is likely to contain similar values again. Therefore, the blocked move from (a,b) to $(a_\star, b_\star)$ has usually a higher acceptance probability than the move from (a,b) to $(a_\star, b)$.

where the Hastings-Ratio H is equal to:

$$H = \frac{p(\boldsymbol{\mu}|\lambda_{\diamond}^2, \lambda_{\star}^2, \sigma^2, \mathbf{\Pi}, \boldsymbol{\delta})}{p(\boldsymbol{\mu}_{\bullet}|\lambda_{\diamond}^2, \lambda_{\star}^2, \sigma^2, \mathbf{\Pi}_{\bullet}, \boldsymbol{\delta}_{\bullet})} \cdot \mathrm{HR}$$

and the factor HR is move-specific (D, A and E):

$$\mathrm{HR}_D = \frac{|\mathbf{\Pi}|}{N_{\star} - |\mathbf{\Pi}_{\bullet}|} \cdot 0.5, \qquad \mathrm{HR}_A = \frac{N_{\star} - |\mathbf{\Pi}|}{|\mathbf{\Pi}_{\bullet}|} \cdot 2, \qquad \mathrm{HR}_E = 1 \tag{13}$$

where $N_{\star}$ is the number of potential covariates, $|.|$ denotes the cardinality, and the factors 2 and 0.5 stem from flipping a coin to determine the type ($\delta \in \{0, 1\}$) of a new covariate X.

Part C - The Markov Chain Monte Carlo (MCMC) algorithm

To generate samples from the posterior distribution in Equation (10), we use a Markov Chain Monte Carlo (MCMC) algorithm, which combines the Gibbs-sampling steps from part A with the Metropolis Hastings steps from part B. We initialize all entities, e.g. $\mathbf{\Pi} = \{\}$, $\boldsymbol{\delta} = (\delta_0) = (0)$, $\boldsymbol{\mu} = \mathbf{0}$, $\lambda_\diamond^2 = 1$, $\lambda_\star^2 = 1$, before we iterate among seven sampling steps:

Gibbs part: Given $\mathbf{\Pi}$ and $\boldsymbol{\delta}$, we re-sample the parameters σ^2, $\boldsymbol{\beta}$, $\lambda_\diamond^2$, $\lambda_\star^2$, and $\boldsymbol{\mu}$. Although the parameters σ^2 and $\boldsymbol{\beta}_B$ can be marginalized out, and thus do not appear in the posterior in Equation (10), the FCDs of $\lambda_\diamond^2$ and $\lambda_\star^2$, and $\boldsymbol{\mu}$ depend on them. Therefore σ^2 and $\boldsymbol{\beta}_B$ have to be sampled too, but they can be withdrawn after sampling step (5). The Gibbs sampling steps have been derived in part A of this supplementary paper. Each step updates one parameter, and the subsequent steps are then always conditional on the newest parameter combination:

(1) $\sigma^{-2}|(\mathbf{y}, \lambda_\diamond^2, \lambda_\star^2, \boldsymbol{\mu}, \mathbf{\Pi}, \boldsymbol{\delta}) \sim GAM\left(a + \frac{T}{2}, b + \frac{1}{2}(\mathbf{y} - \mathbf{X}_B\tilde{\boldsymbol{\mu}})^\mathsf{T}\sigma^{-2}[\mathbf{I} + \mathbf{X}_B\tilde{\mathbf{\Sigma}}\mathbf{X}_B^\mathsf{T}]^{-1}(\mathbf{y} - \mathbf{X}_B\tilde{\boldsymbol{\mu}})\right)$

(2) $\boldsymbol{\beta}_B|(\sigma^2, \lambda_\diamond^2, \lambda_\star^2, \boldsymbol{\mu}, \mathbf{\Pi}, \boldsymbol{\delta}) \sim \mathcal{N}_U\left([\tilde{\mathbf{\Sigma}}^{-1} + \mathbf{X}_B^\mathsf{T}\mathbf{X}_B]^{-1}(\tilde{\mathbf{\Sigma}}^{-1}\tilde{\boldsymbol{\mu}} + \mathbf{X}_B^\mathsf{T}\mathbf{y})\ ,\ \sigma^2[\tilde{\mathbf{\Sigma}}^{-1} + \mathbf{X}_B^\mathsf{T}\mathbf{X}_B]^{-1}\right)$
where $U = \sum_{i=1}^{|\mathbf{\Pi}|+1} (\delta_i + K \cdot (1 - \delta_i))$.

(3) $\lambda_\diamond^{-2}|(\sigma^2, \boldsymbol{\beta}_B, \lambda_\star^2, \boldsymbol{\mu}, \mathbf{\Pi}, \boldsymbol{\delta}) \sim GAM\left(\alpha_\diamond + \frac{Kn_2}{2}, \beta_\diamond + \frac{1}{2}\sigma^{-2}\sum_{k=1}^{K}(\boldsymbol{\beta}_k - \boldsymbol{\mu})^\mathsf{T}(\boldsymbol{\beta}_k - \boldsymbol{\mu})\right)$

(4) $\lambda_\star^{-2}|(\sigma^2, \boldsymbol{\beta}_B, \lambda_\diamond^2, \boldsymbol{\mu}, \mathbf{\Pi}, \boldsymbol{\delta}) \sim GAM\left(\alpha_\star + \frac{n_1}{2}, \beta_\star + \frac{1}{2}\sigma^{-2}\boldsymbol{\beta}_\star^\mathsf{T}\boldsymbol{\beta}_\star\right)$

(5) $\boldsymbol{\mu}|(\lambda_\diamond^2, \lambda_\star^2, \sigma^2, \mathbf{\Pi}, \boldsymbol{\delta}) \sim \mathcal{N}_V(\boldsymbol{\mu}^\ddagger, \mathbf{\Sigma}^\ddagger)$; see Equations (6-8), where
$V = \sum_{i=1}^{|\mathbf{\Pi}|+1} (1 - \delta_i)$ is the number of covariates with component-specific coefficients.

Metropolis-Hastings part: Withdraw σ^2 and $\boldsymbol{\beta}_B$, and keep $\lambda_\diamond^2$, $\lambda_\star^2$ and $\boldsymbol{\mu}$. Perform the two blocked Metropolis-Hastings moves from part B:

(6) We propose to replace $[\boldsymbol{\delta}, \boldsymbol{\mu}]$ by $[\boldsymbol{\delta}_\bullet, \boldsymbol{\mu}_\bullet]$, and we accept the new pair with the probability given in Equation (11). If accepted, we replace $[\boldsymbol{\delta}, \boldsymbol{\mu}]$ by $[\boldsymbol{\delta}_\bullet, \boldsymbol{\mu}_\bullet]$, otherwise we leave $[\boldsymbol{\delta}, \boldsymbol{\mu}]$ unchanged.

(7) We propose to replace $[\mathbf{\Pi}, \boldsymbol{\delta}, \boldsymbol{\mu}]$ by $[\mathbf{\Pi}_\bullet, \boldsymbol{\delta}_\bullet, \boldsymbol{\mu}_\bullet]$, and we accept the new triple with the probability given in Equation (12). If accepted, we replace $[\mathbf{\Pi}, \boldsymbol{\delta}, \boldsymbol{\mu}]$ by $[\mathbf{\Pi}_\bullet, \boldsymbol{\delta}_\bullet, \boldsymbol{\mu}_\bullet]$, otherwise we leave $[\mathbf{\Pi}, \boldsymbol{\delta}, \boldsymbol{\mu}]$ unchanged.

The MCMC algorithm generates a posterior sample:

$$\{\mathbf{\Pi}^{(w)}, \boldsymbol{\delta}^{(w)}, \lambda_{\star,(w)}^2, \lambda_{\diamond,(w)}^2, \boldsymbol{\mu}^{(w)}\} \sim p(\mathbf{\Pi}, \boldsymbol{\delta}, \lambda_\star^2, \lambda_\diamond^2, \boldsymbol{\mu}|\mathbf{y}) \qquad (w = 1, \ldots, W) \tag{14}$$

As described in the main paper, we run the MCMC algorithm for $W = 100,000$ ($W = 100k$) iterations. We set the burn-in phase to $50k$ and we sample every 100th graph during the sampling phase. This yields $R = 500$ posterior samples for each response Y.

Part D - Gaussian Process smoothing

In this part of the supplementary paper we provide additional results for the proposed Gaussian process smoothing method; see Section 2.5 of the main paper. The proposed GP smoothing method can be implemented in 4 ways:

1. The GP parameters can either be sampled via Markov Chain Monte Carlo simulations ('**MCMC**') **or** their maximum a posteriori estimates ('**MAP**') can be computed.

2. Given the GP parameters, the required response vectors $y_{k,\star}$ can either be sampled from Equation (11) of the main paper ('**PRED-SAMPLING**') **or** $y_{k,\star}$ can be set to its predictive expectation, $\hat{y}_{k,\star}$, see Equation (12) of the main paper ('**PRED-EXPECTATION**').

For our study, we implemented all four combinations:

- **MAP and PRED-EXPECTATION** (used in the main paper)
- **MAP and PRED-SAMPLING**
- **MCMC and PRED-EXPECTATION**
- **MCMC and PRED-SAMPLING**

We compute the MAP estimates of the GP parameters (σ^2, ξ^2 and l) using the Matlab package 'GPstuff' (Vanhatalo *et al.*, 2013). For MCMC sampling of the GP parameters (σ^2, ξ^2 and l) we implemented three Metropolis-Hastings moves. In the i-th move ($i = 1, 2, 3$) a small random number, uniformly distributed on $[-\epsilon, \epsilon]$, is added to the current instantiation of the i-th GP parameter. (We used $\epsilon = 0.01$.) This yields a new candidate value for parameter i. If positive, the new candidate parameter is accepted with the Metropolis-Hastings acceptance probability. If the new candidate parameter is negative or not accepted, the i-th parameter is left unchanged. As initial values for our MCMC sampling scheme we use the MAP estimates from 'GPstuff'.

Additional results for synthetic data:

In Section 4.1 of the main paper, the GP smoothing method is applied to synthetic autoregressive (AR) and moving average (MA) data. As supplement to Figure 2 of the main paper, we here show and compare the performances of the four GP approaches. Figure 1 shows the results for the AR data, and Figure 2 shows the results for the MA data. In both figures the top left panel refers to the results of: 'MAP and PRED-EXPECTATION'. Those results are also shown in Figure 2 of the main paper.

Figures 1-2 show that the four GP variants perform similar and yield comparable covariate scores. The two true covariates X_1 and X_2 yield higher scores and can clearly be distinguished from the irrelevant variables $X_3, \ldots, X_8$. Tables 1-2 provide the method-specific average scores of the true covariates and the average scores of the irrelevant variables. Although neither significant nor substantial, the method 'MAP and PRED-EXPECTATION', used in the main paper, appears to perform slightly better than the other three GP methods: The difference between the average scores of the true and the false variables is slightly higher.

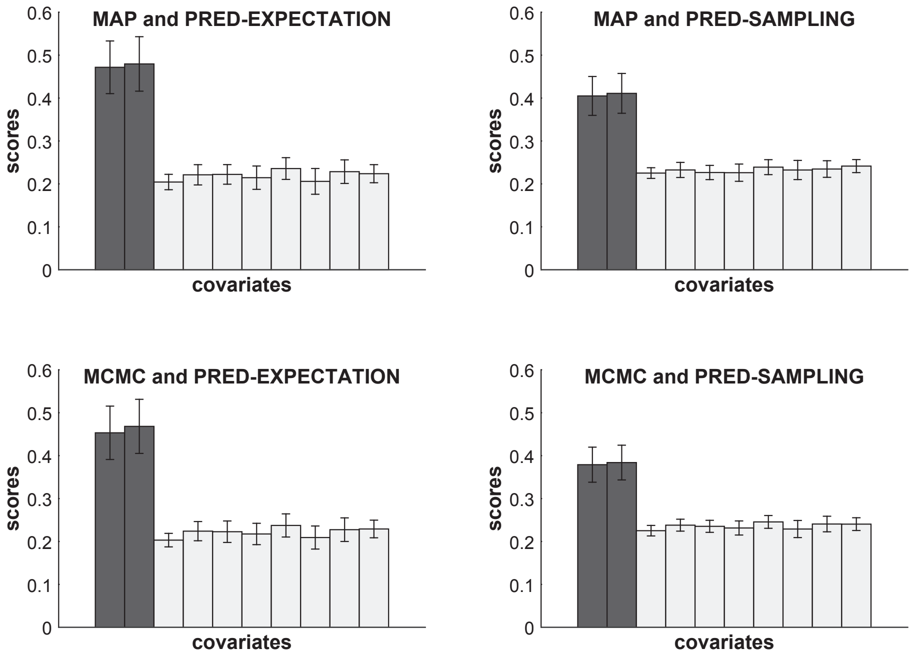

Figure 1: Average covariate scores for **auto-regressive** (**AR**) data. There is a histogram for each of the four GP smoothing methods. The dark grey bars refer to the scores of the two true covariates, and the light grey bars refer to the irrelevant variables. The error bars indicate standard deviations. The top left histogram corresponds to the top right histogram in Figure 2 of the main paper.

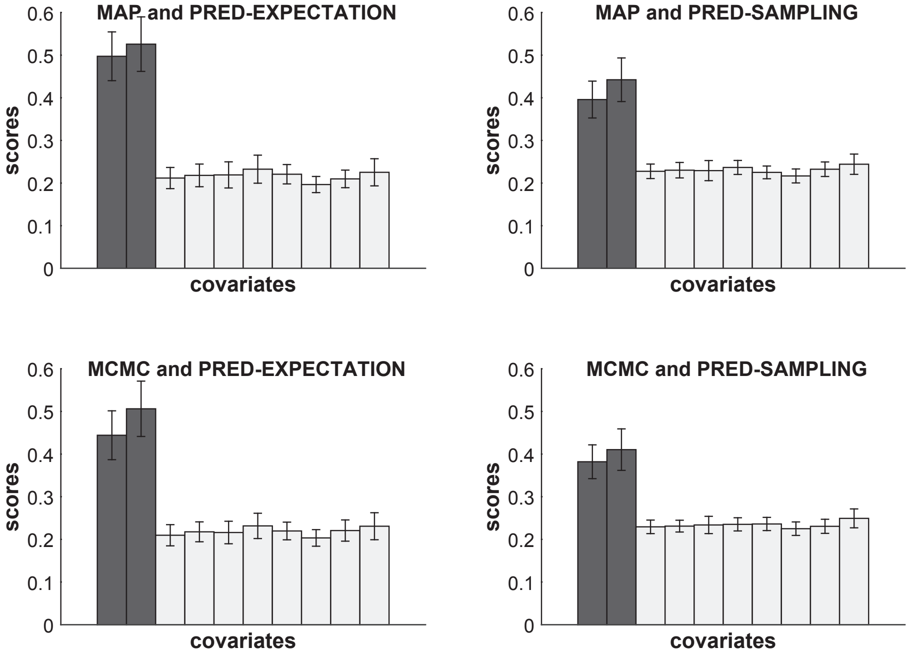

Figure 2: Average covariate scores for **moving-average** (**MA**) data. There is a histogram for each of the four GP smoothing methods. The dark grey bars refer to the scores of the two true covariates, and the light grey bars refer to the irrelevant variables. The error bars indicate standard deviations. The top left histogram corresponds to the bottom right histogram in Figure 2 of the main paper.

	avg. scores true	avg. scores false	difference
without GP	0.2742	0.2589	0.0153
MAP and PRED-EXPECTATION	0.4756	0.2195	**0.2561**
MAP and PRED-SAMPLING	0.4079	0.2323	0.1757
MCMC and PRED-EXPECTATION	0.4607	0.2214	0.2394
MCMC and PRED-SAMPLING	0.3814	0.2357	0.1457

Table 1: Summary of results for **auto-regressive** (**AR**) data. For each GP method, the table gives the average scores of the two true covariates and the average scores of the eight false variables. The last column gives the differences of the averages.

	avg. scores true	avg. scores false	difference
without GP	0.2706	0.2609	0.0104
MAP and PRED-EXPECTATION	0.5115	0.2165	**0.2948**
MAP and PRED-SAMPLING	0.4191	0.2276	0.1888
MCMC and PRED-EXPECTATION	0.4752	0.2163	0.2565
MCMC and PRED-SAMPLING	0.3962	0.2318	0.1624

Table 2: Summary of results for **moving-average** (**MA**). For each GP method, the table gives the average scores of the two true covariates and the average scores of the eight false variables. The last column gives the differences of the averages.

Additional results for mTORC1 data:

In Section 4.5 of the main paper, the new partially non-homogeneous DBN model was combined with the GP-method: 'MAP and PRED-EXPECATION' to reconstruct the mTORC1 network topology. We also inferred the mTORC1 network using our new model in combination with the three alternative GP-methods, and computed the GP-method-specific edge scores.

Figure 3 shows scatter plots, in which the scores of the reference method: 'MAP and PRED-EXPECATION' (vertical axes) are plotted against those of the three alternative GP methods (horizontal axes). Table 3 lists all edges that have been inferred and indicates, with which of the GP-methods those edges were extracted.

From the scatter plots it can be seen that the method-specific edges scores are correlated (Pearson correlation coefficients: $\rho = 0.86$, $\rho = 0.84$, and $\rho = 0.73$), and Table 3 shows that the four networks share seven edges. Moreover, the networks for 'MAP and EXPECTATION' and 'MCMC and EXPECTATION' are similar (three mismatches), and the networks for 'MAP and SAMPLING' and 'MCMC and SAMPLING' are similar (also three mismatches). We conclude that it does not make a large difference, here, whether the GP parameters are MCMC sampled or MAP estimated.

It can also be seen from Table 3 that the networks of the latter group ('... and SAMPLING') have less edges (they lose 5 edges and gain 1-2 edges). Further diagnostics (not shown) suggest that the sparsity of the networks can be explained as follows: The mTORC1 data are rather sparse ($T_k = 10$ data points for each of $K = 2$ conditions only), so that there is a high amount of uncertainty in the predictive curves. The predictive distributions (see Equation (11) of the main paper) are therefore rather diffuse with high variances of the individual elements. Sampling from diffuse predictive distributions, yields response curves that can vary substantially from MCMC

iteration to MCMC iteration. For the '...and SAMPLING' approaches the edge scores are now averages across all possible response vectors (with weights being their predictive probabilities), leading to lower edges scores and, hence, to sparser network topologies.

It is beyond the scope of our paper to systematically cross-compare the four GP-methods, but our results on sparse mTORC1 data suggest:

- It does not make a large difference whether the GP parameters are MCMC sampled or MAP estimated. Both approaches yield similar results.
- But it does make a difference whether the response vectors are sampled from the predictive distribution or set equal to the predictive expectation, cf. Equations (11-12) of the main paper.
- We observed uncertainty in the predictive distributions, so that sampling from the predictive distribution leads to lower edge scores and correspondingly sparser network topologies.
- We would conclude that sampling from the predictive distribution is therefore a more conservative (=robust) approach.
- We would further argue that using the most likely curve (i.e. setting the curve to the predictive expectation), as done in the main paper, is appropriate if the main goal is to generate hypotheses about new network interactions.

			MAP and EXPECTATION	MAP and SAMPLING	MCMC and EXPECTATION	MCMC and SAMPLING
IRS1-pS636	→	p70-S6K-pT389	**X**	**X**	**X**	**X**
p70-S6K-pT389	→	IRS1-pS636	**X**	**X**	**X**	**X**
IRS1-pS636	→	mTOR-pS2448	**X**	**X**	**X**	**X**
AKT-pS473	→	AKT-pT	**X**	**X**	**X**	**X**
AKT-pT	→	AKT-pS473	**X**	**X**	**X**	**X**
AKT-pS473	→	PRAS40-pT246	**X**	**X**	**X**	**X**
PRAS40-pT246	→	PRAS40-pS183	**X**	**X**	**X**	**X**
mTOR-pS2448	→	TSC2-pS1387	**X**	-	**X**	-
IRS1-pS636	→	AKT-pT308	**X**	-	**X**	-
PRAS40-pS183	→	mTOR-pS2481	**X**	-	-	-
p70-S6K-pT389	→	PRAS40-pS183	**X**	-	**X**	-
AKT-pT308	→	PRAS40-pT246	**X**	-	**X**	-
IR-beta-pY1146	→	AKT-pT308	-	**X**	-	-
PRAS40-pT246	→	AKT-pS473	-	-	**X**	**X**
TSC2-pS1387	→	mTOR-pS2481	-	-	**X**	-
PRAS40-pT246	→	AKT-pT308	-	-	-	**X**

Table 3: Overview of the 16 inferred mTORC1 network edges. The four predicted mTORC1 networks share 7 edges, but there are mismatches for 9 edges. The table has a row for each of the 16 edges, and a column for each GP-method. The cells indicate if the GP-method extracted the corresponding edge ('**X**') or not ('-').

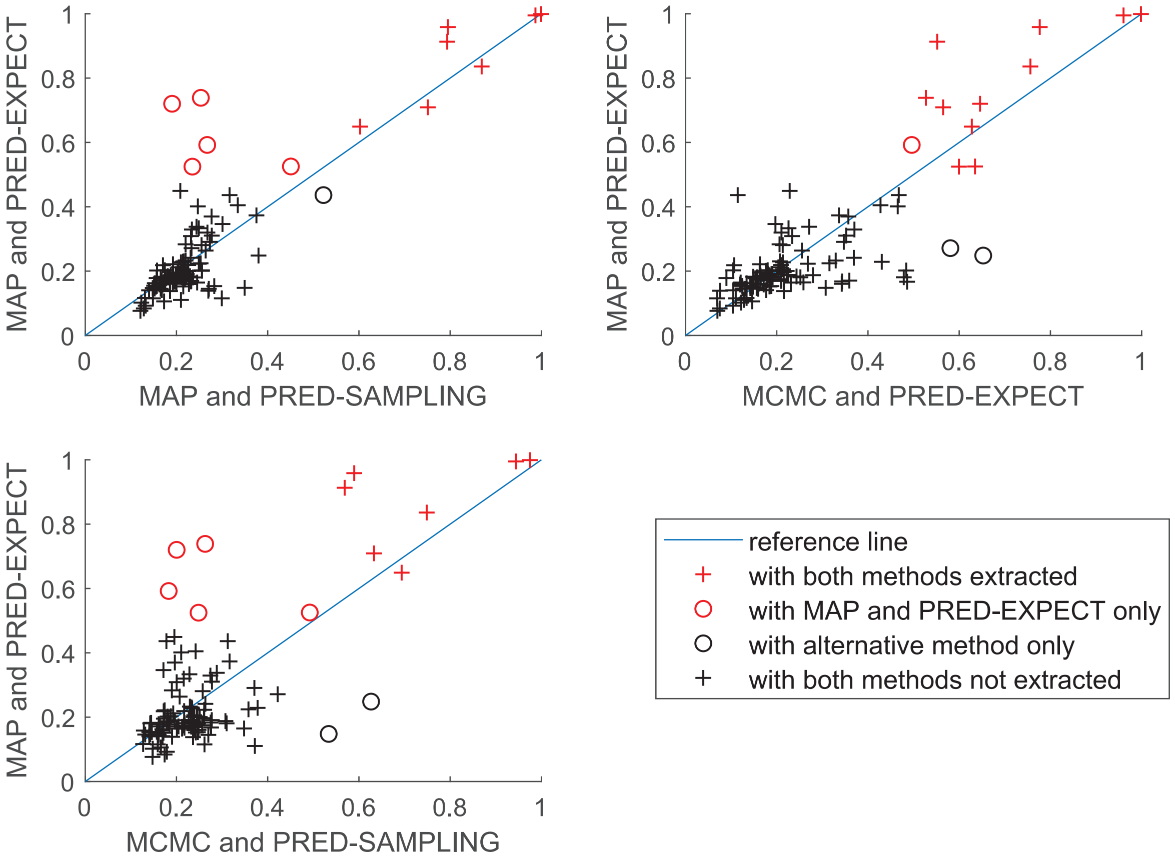

Figure 3: Comparison of GP-method-specific edge scores for the mTORC1 data. We inferred the mTORC1 network topology with our new partially non-homogeneous DBN, using four different GP-methods. For each GP-method we computed the method-specific edge scores. We then plotted the edges scores of the reference method, used in the main paper, 'MAP and PRED-EXPECTATION' against those of the three competing methods: vs. MAP and PRED-SAMPLING (top, left), vs. MCMC and PRED-EXPECTATION (top, right), and vs. MCMC and PRED-SAMPLING (bottom, left). The '+' symbol indicates edges, onto which both methods agree. The 'o' symbol indicates edges that are only inferred with one of the two GP methods.

Part E - The topology of the RAF signalling pathway

Figure 4 show the topology of the RAF signalling pathway, as reported in Sachs *et al.* (2005). The RAF pathway consists of $N = 11$ nodes (phosphorylated proteins) and possesses 20 directed edges. We used this network topology for generating synthetic network data. See Section 4.2 of the main paper for further details.

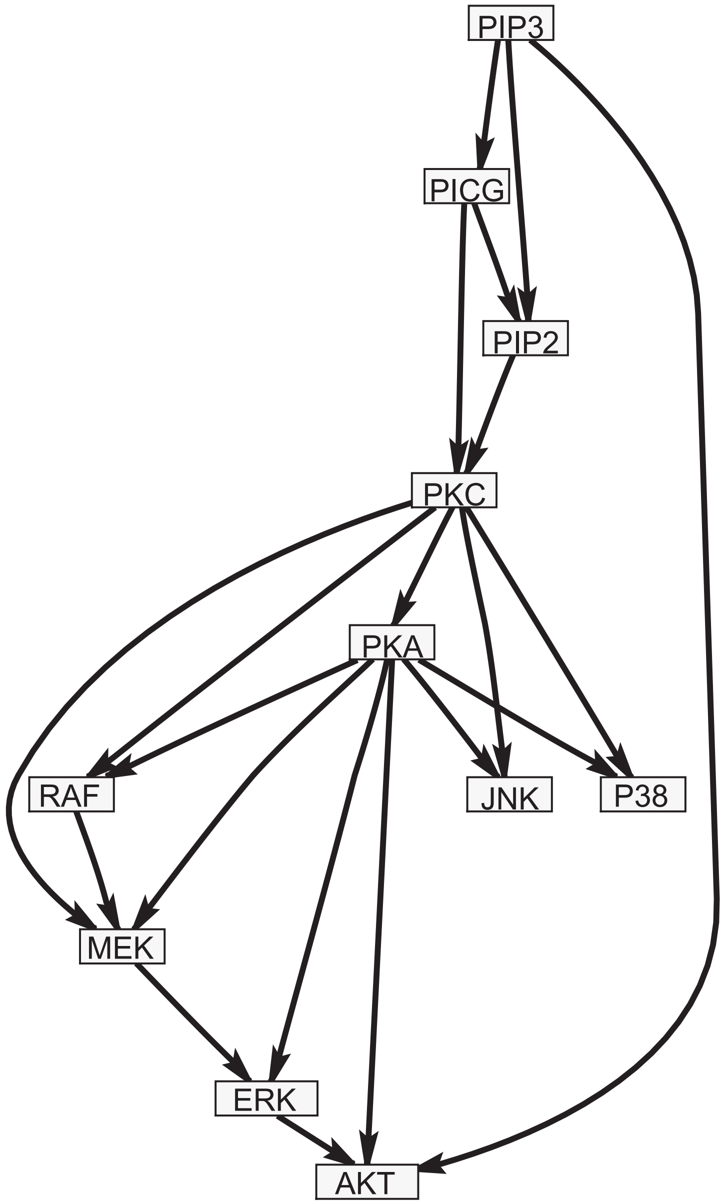

Figure 4: **The topology of the RAF signalling pathway, as reported in Sachs *et al.* (2005).**

Part F - Scalability and convergence analysis (additional results)

In this part of the supplementary material we provide additional figures for Section 4.3 of the main paper. Figures 5-7 show superimposed edge score scatter plots for the network sizes $N = 10$, $N = 25$ and $N = 50$. These figures correspond to the left panel of Figure 4 (main paper). The top right panel of Figure 4 (main paper) shows average AUC trace plots. Figure 8 of this supplement shows the individual AUC trace plots, from which the average curves in the top right panel of Figure 4 (main paper) were computed. We refer to these figures in Section 4.3 of the main paper.

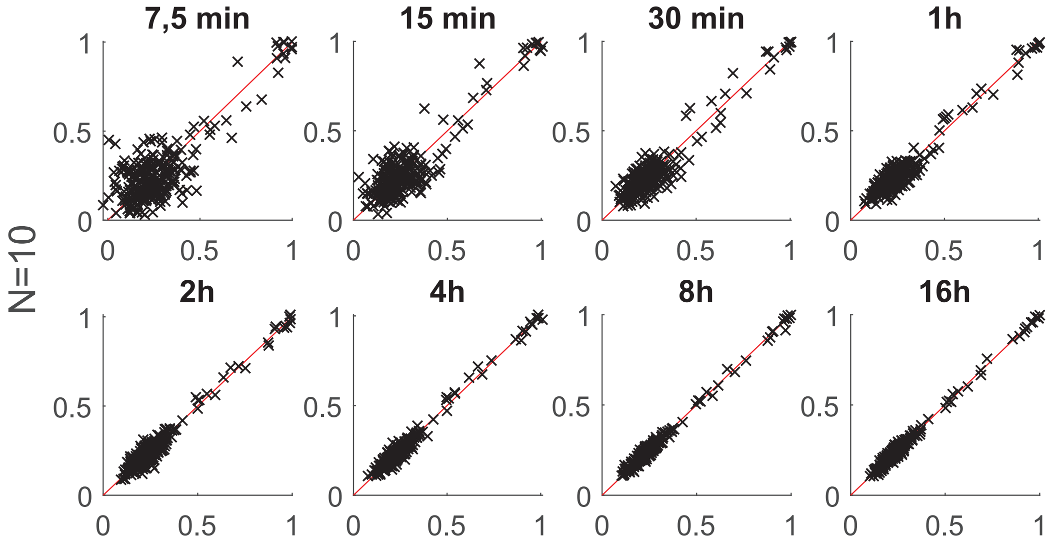

Figure 5: Superimposed scatter plots for networks with $N = 10$ nodes, after different computational times. See caption of Figure 4 in the main paper for further details.

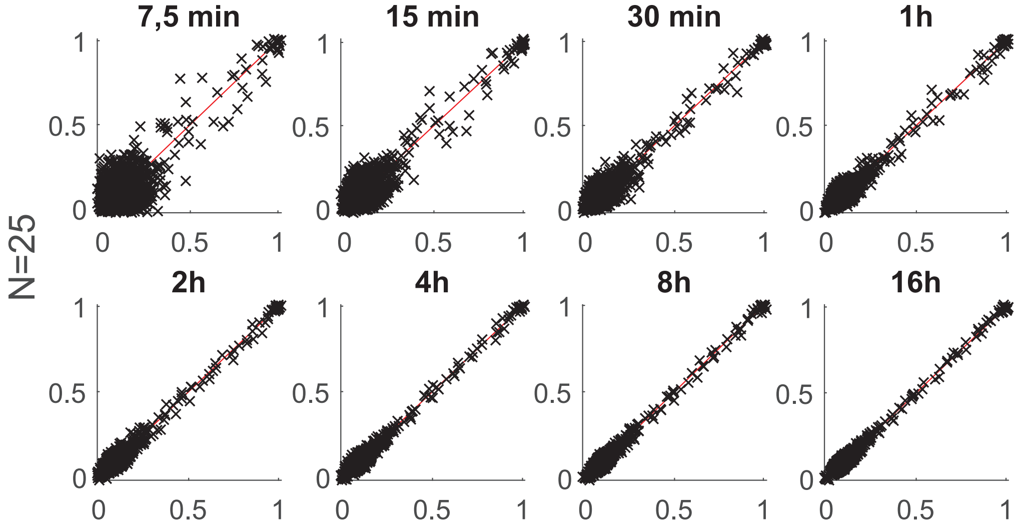

Figure 6: Superimposed scatter plots for networks with $N = 25$ nodes, after different computational times. See caption of Figure 4 in the main paper for further details.

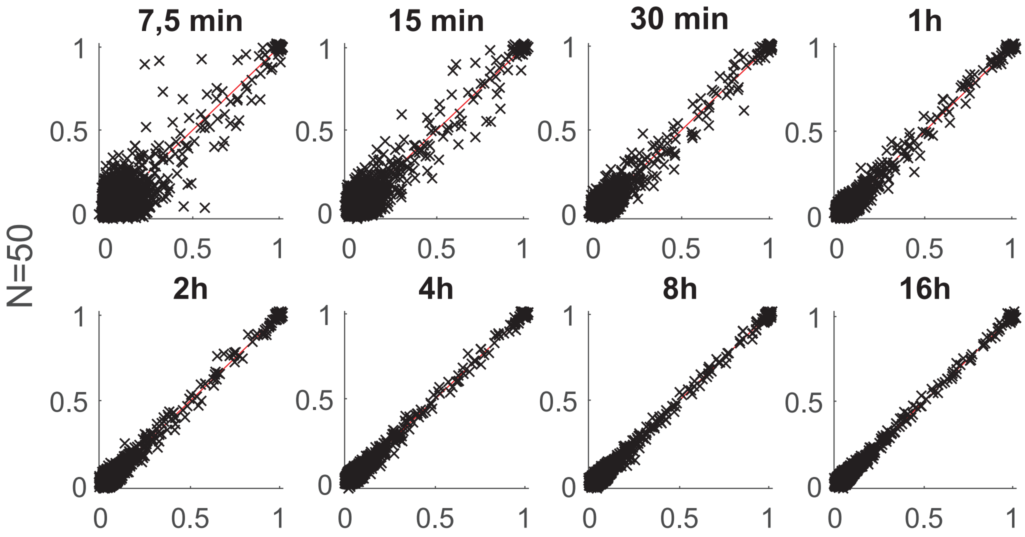

Figure 7: Superimposed scatter plots for networks with $N = 50$ nodes, after different computational times. See caption of Figure 4 in the main paper for further details.

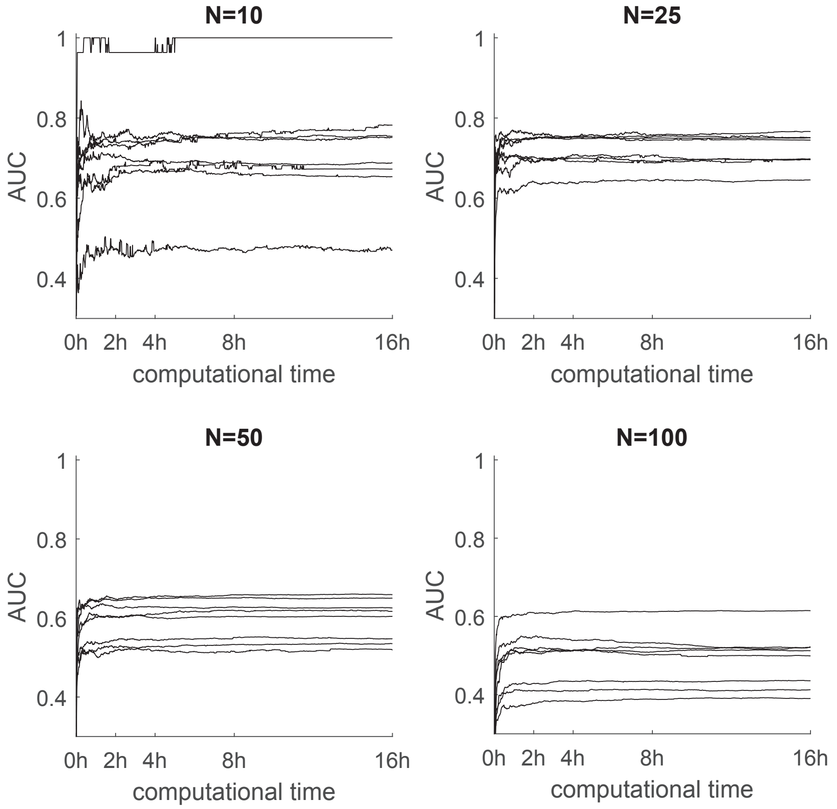

Figure 8: Precision-recall AUC trace plots: There is a panel for each network size $N \in \{10, 25, 50, 100\}$ and each panel shows the individual AUC trace plots for 10 data sets. The AUC trace plots in the top right panel of Figure 4 (main paper) are the average curves.

References

Bishop, C. M. (2006). *Pattern Recognition and Machine Learning*. Springer, Singapore.

Green, P. (1995). Reversible jump Markov chain Monte Carlo computation and Bayesian model determination. *Biometrika*, **82**, 711–732.

Liang, F., Liu, C., and Carroll, R. (2010). *Advanced Markov chain Monte Carlo methods: Learning from past samples*. Wiley Series in Computational Statistics. John Wiley and Sons, Cornwall, UK.

Sachs, K., Perez, O., Pe'er, D., Lauffenburger, D., and Nolan, G. (2005). Causal protein-signaling networks derived from multiparameter single-cell data. *Science*, **308**, 523–529.

Vanhatalo, J., Riihimäki, J., Hartikainen, J., Jylänki, P., Tolvanen, V., and Vehtari, A. (2013). GPstuff: Bayesian modeling with Gaussian processes. *The Journal of Machine Learning Research*, **14**(1), 1175–1179.
